# Megathrust earthquake drives drastic organic carbon supply to the hadal trench

**DOI:** 10.1038/s41598-019-38834-x

**Published:** 2019-02-07

**Authors:** A. Kioka, T. Schwestermann, J. Moernaut, K. Ikehara, T. Kanamatsu, C. M. McHugh, C. dos Santos Ferreira, G. Wiemer, N. Haghipour, A. J. Kopf, T. I. Eglinton, M. Strasser

**Affiliations:** 1 Institute of Geology, University of Innsbruck, Innsbruck, Austria; 2 Geological Survey of Japan, National Institute of Advanced Industrial Science and Technology (AIST), Tsukuba, Japan; 3 R&D Center for Earthquake and Tsunami, Japan Agency of Marine Science and Technology (JAMSTEC), Yokosuka, Japan; 4 Earth and Environmental Sciences, Queens College, City University of New York, New York, USA; 5 MARUM – Center for Marine Environmental Sciences, University of Bremen, Bremen, Germany; 6 Geological Institute, ETH Zürich, Zürich, Switzerland; 7 Laboratory of Ion Beam Physics, ETH Zürich, Zürich, Switzerland

## Abstract

The giant 2011 Tohoku-oki earthquake has been inferred to remobilise fine-grained, young surface sediment enriched in organic matter from the slope into the >7 km deep Japan Trench. Yet, this hypothesis and assessment of its significance for the carbon cycle has been hindered by limited data density and resolution in the hadal zone. Here we combine new high-resolution bathymetry data with sub-bottom profiler images and sediment cores taken during 2012–2016 in order to map for the first time the spatial extent of the earthquake-triggered event deposit along the hadal Japan Trench. We quantify a sediment volume of ~0.2 km^3^ deposited from spatially-widespread remobilisation of young surficial seafloor slope sediments triggered by the 2011 earthquake and its aftershock sequence. The mapped volume and organic carbon content in sediment cores encompassing the 2011 event reveals that this single tectonic event delivered >1 Tg of organic carbon to the hadal trench. This carbon supply is comparable to high carbon fluxes described for other Earth system processes, shedding new light on the impact of large earthquakes on long-term carbon cycling in the deep-sea.

## Introduction

Hadal trenches (6–11 km water depth)^1^, such as those formed by the downward bending of oceanic crust along subduction plate boundaries, are among the least explored environments on Earth. Hadal benthic communities are subject to extreme hydrostatic pressure, and thus host many unique piezophilic organisms^1,2^. The steep slopes and isolated nature of the ultra-deep basins facilitate the accumulation of organic matter in hadal trenches. In addition, sediment mass-wasting events and accompanying sinking of organic particles can supply relatively fresh organic matter to the hadal trenches^3,4^, which can support a high level of microbial activity in this secluded habitat^5^. On longer geological time-scales, the deposition, burial, and eventual subduction of organic carbon (OC) – rich marine sediments in plate subduction zones can play a key role in the earth’s long-term carbon cycle, and influence atmospheric CO_2_ concentrations over millions of years^6–8^. Despite the growing understanding of pivotal role of the carbon cycle in governing the Earth’s climate and biosphere, the nature and quantification of carbon supply to hadal environments remains poorly constrained.

Recently, Bao *et al*.^9^ reported sediment and OC export to the hadal zone of the Japan Trench triggered by large subduction zone earthquakes, such as the moment magnitude (*M*_*w*_) 9 Tohoku-oki earthquake in 2011 – the third largest instrumentally recorded earthquake worldwide. This event also included large coseismic slip breaching the hadal trench^10–13^. Submarine video observations and sediment cores document earthquake- and tsunami-related remobilisation of surficial sediment enriched in organic matter and deposition from the slope to the more than 7 km deep Japan Trench^4,9,14–20^. The resulting submarine homogeneous muddy-flow deposits have been shown to be highly enriched in short-lived excess ^210^Pb, implying shallow (only centimetres-deep) but widespread sediment remobilisation extending over large areas^18^. However, evidence for a link between earthquake-triggered remobilisation of surficial sediment and carbon export to the hadal trench is based on only a few cores from the central part of the Japan Trench^4,9^. Until now, the absence of high-resolution spatial mapping of the entire 2011 event deposits over the complete along-strike extent of the hadal trench has hampered quantification of sediment volume and OC content, and hence the assessment of its role and significance on the sediment budget and carbon cycle.

**Figure 1:**
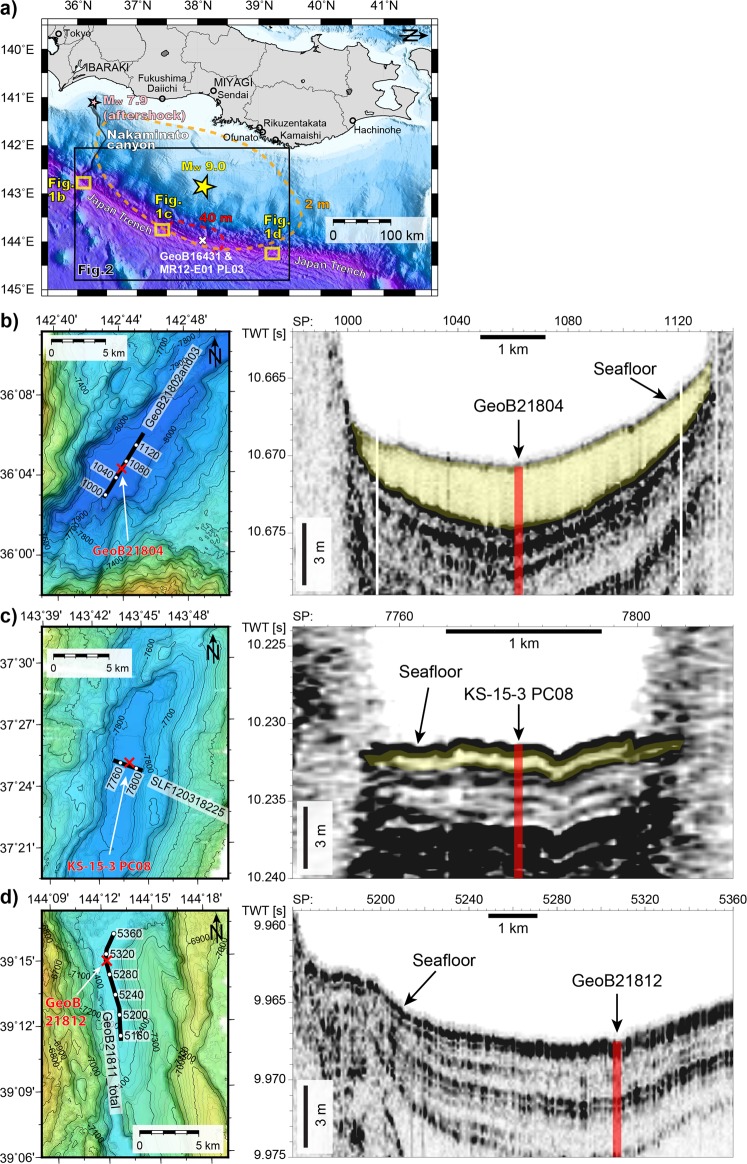
(**a**) Location map of study area with the epicentre of the 2011 *M*_*w*_9.0 Tohoku-oki earthquake (yellow star; 142°52′E, 38°6′N from Japan Meteorological Agency (JMA), http://www.data.jma.go.jp/svd/eqdb/data/shindo/), the epicentre of the largest aftershock (*M*_*w*_7.9) occurring 30 minutes after the *M*_*w*_9.0 mainshock (pink star; 141°7′E, 36°17′N from USGS, https://earthquake.usgs.gov/earthquakes/eventpage/usp000hvpa/), and co-seismic slip distribution (dashed lines) of the *M*_*w*_9.0 mainshock. The dashed lines represent 40 m- (red)^21^ and 2 m-coseismic slip (orange)^22^. (**b**) Bathymetric map with a track line of studied SBP in a trench-fill basin in the southern Japan Trench and a PARASOUND SBP line along the basin, imaging a thick acoustically-transparent body with ponding geometries, interpreted as the 2011 event (yellow transparent layers). (**c**) Bathymetric map with a track line of studied SBP in a fill-basin in the central Japan Trench and a noise-attenuated PARASOUND SBP line across the basin, imaging an intermediately thick 2011 event deposit. (**d**) Bathymetric map with a track line of studied SBP in a fill-basin in the northern part of study area where the SBP does not image a 2011 event deposit.

Here, we present newly-acquired high-resolution bathymetry data of the entire trench axis, along with >800 km of high-resolution subbottom profiler (SBP) data acquired during 2012–2016 along the Japan Trench between 36°N and 39.5°N (i.e., the extent roughly corresponding to the large coseismic slip of the 2011 earthquake^21,22^ (Fig. 1a)). These data allow, for the first time, imaging and spatial mapping of the full extent of 2011 event deposit along the entire Japan Trench. When combined with radionuclide dating and total organic carbon (TOC) measurements on sediment cores, we estimate the total volume of fine-grained surficial sediment remobilised by the 2011 earthquake and the mass of associated OC deposited in the trench.

## The 2011 Event Deposit in the Hadal Trench

**Figure 2:**
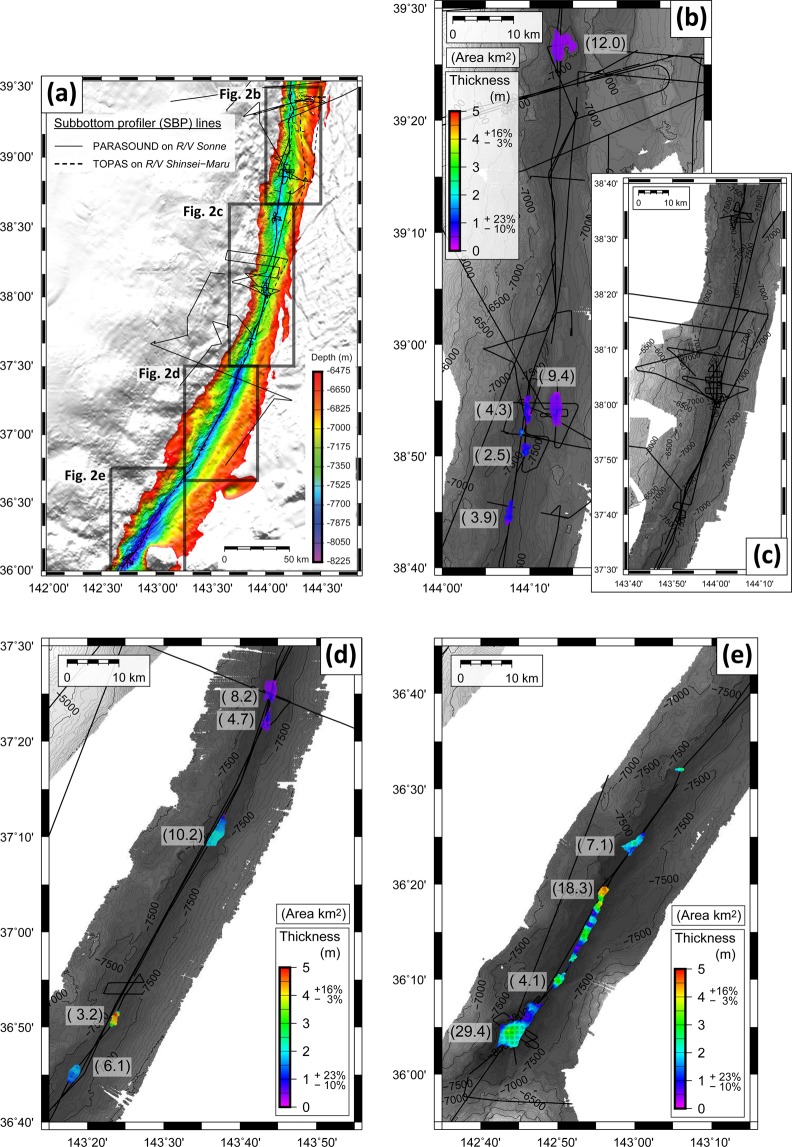
(**a**) Bathymetry map with studied SBP lines in the studied Japan Trench area. (**b**) Thickness map of the 2011 event deposit identified by SBP mapping with the extent area (km^2^) in the central Japan Trench (38°40′–39°30′N). Black lines are track lines of studied SBP data. (**c**) Thickness map in the central Japan Trench (37°30′–38°40′N) where the 2011 event deposit is not identified in the studied SBP data. (**d**) Thickness map in the southern Japan Trench (36°40′–37°30′N) and (**e**) southernmost Japan Trench (35°55′–36°45′N). The errors in thickness (shown right of colour bar) result from uncertainties in the choice of internal velocity and the vertical resolution of studied SBP data (See Supplementary Information). Note that only the numbers of extent area of the 2011 event deposit exceeding 1 km^2^ are shown.

Our high-resolution bathymetric data clearly image the N-S to NNW-SSE trending horst-and-graben structures formed by flexural bending of the subducting Pacific plate. This results in relatively rough trench-floor morphology with 0.5–15 km long and 0.5–5 km wide isolated trench-fill and graben-fill basins, with different along-strike connectivity (Figs 1, 2, Supplementary Figs 1–12). The trench-floor along its southernmost segment is as deep as 8,030 m, while water depth in the central trench ranges between 7,400 m and 7,700 m. Trench-basins in the southern Japan Trench tend to be comparatively larger than those in the central trench. In particular, the trench around 38°N is narrow and only comprises very small basins with limited lateral connectivity as a consequence of local slumps and trench-floor deformation by coseismic slip-to-the trench^12,15^.

**Figure 3:**
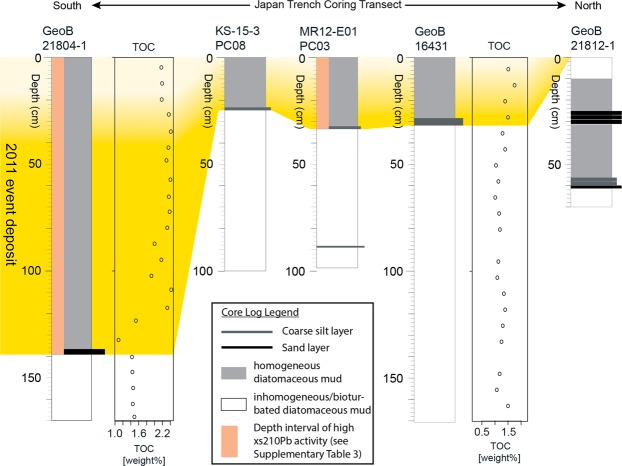
Correlation of cores taken along the trench with radio-nuclide dating, and total organic carbon (TOC) content. Cores MR12-E01 PL03, GeoB16431, and KS-15-3 PC08 are from published data^9,17,20^.

High-resolution SBP data across flat trench-basins image distinct, acoustically-transparent bodies with ponding geometries (Fig. 1), that occur as the uppermost acoustic mappable unit immediately below the seafloor reflector (see “Methods”), suggesting very recent event-deposition of homogeneous, fine-grained sediments (c.f. ref.^23^). The acoustic facies of these depositional bodies are distinct from chaotic reflection patterns in areas of rough seafloor where local deep-seated slumps have transported older sedimentary units into trench basins^15,24^. The interpretation of the recent deposition of fine-grained remobilised sediment in SBP data is validated by published core data around 38°N (ref.^17,20^) and our new cores in the south (core GeoB21804; positive evidence) and north (core GeoB21812; absence of evidence) (Fig. 3). The cores document homogeneous diatomaceous mud deposits overlying a thin oxidized layer, implying very recent burial of the oxidised water-sediment interface by the mass deposition. Short-lived radionuclide data^4,17^ demonstrate that these deposits must be related to recent resedimentation events linked to the 2011 earthquake (Fig. 3). High excess (unsupported) ^210^Pb activity throughout most of the thick event deposit cored in the southern-most trench-fill basin (core GeoB21804; Fig. 3 and Supplementary Table 3) now allows along-strike correlation of the 2011-event deposit of the entire trench. Notably, this finding also implies that the source of the sediment mass comprising the acoustically-transparent bodies (*i*) must result from earthquake-triggered remobilisation of surface-most slope sediments (i.e., the upper few centimetres of surficial sediment remobilised over large areas of the seafloor (c.f. ref.^18,25^) and (*ii*) does not comprise significant amounts of older sediment derived from locally remobilised by earthquake-triggered slumps^15,24^.

**Figure 4:**
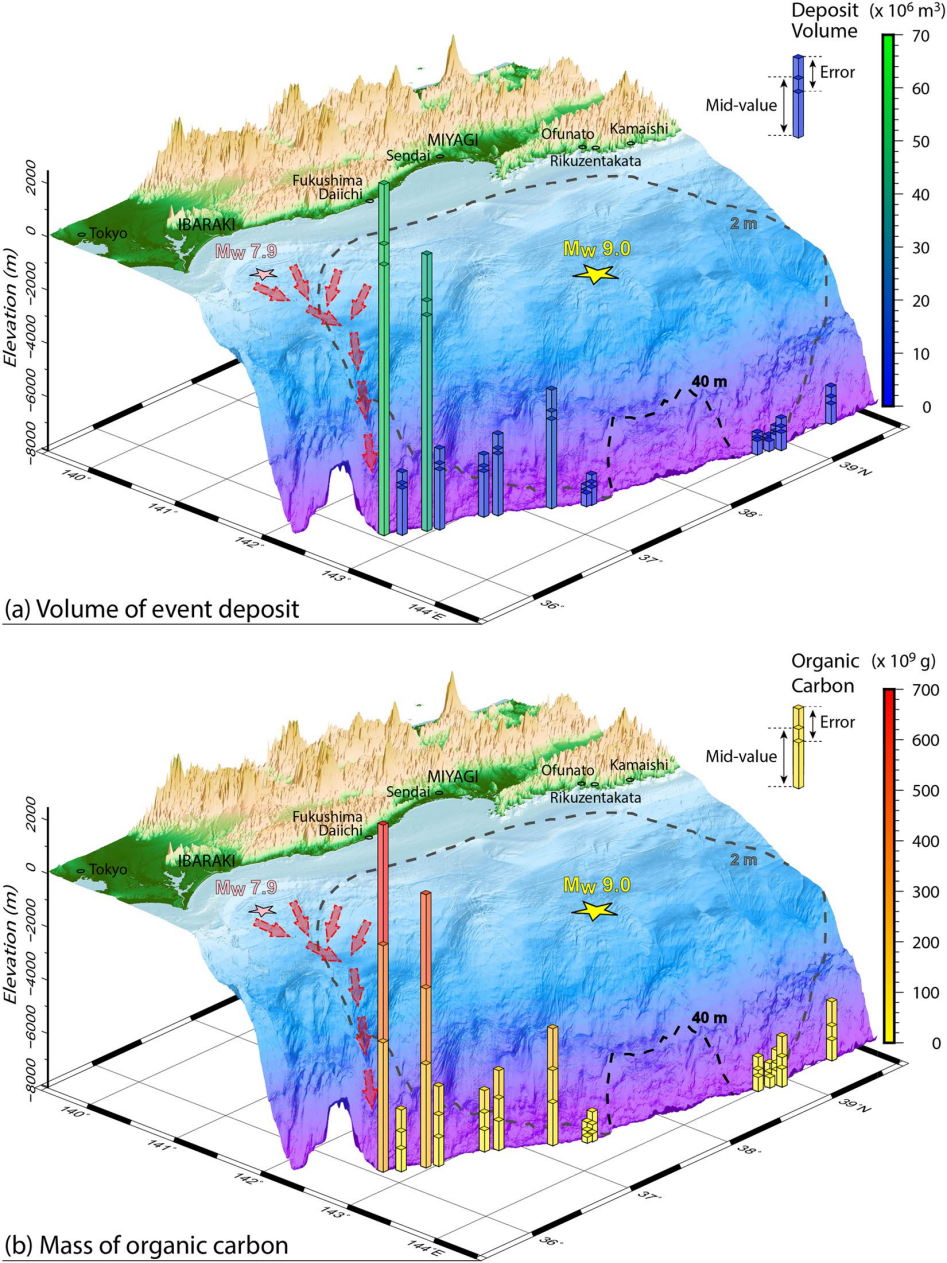
(**a**) Volume of the 2011 event deposit in the studied fill-basins over the central and southern Japan Trench (Supplementary Table 1). (**b**) Mass of organic carbon (OC) contained within the 2011 event deposit in the studied fill-basins. Only the volumes and OC masses of the 2011 event deposits extending over the areas of >1 km^2^ identified by SBP data (Fig. 2) are shown. The dashed lines are coseismic slip of the *M*_*w*_9.0 mainshock of 40 m (black)^21^ and 2 m (gray)^22^. The 3D elevation is made using data from *R/V Sonne* SO251-1 cruise, JAMSTEC-DARWIN database, and GEBCO_2014 Grid^48^.

We spatially mapped the extensive, acoustically-transparent 2011-event deposits, as imaged by SBP in 19 basins in the southern (36.0°–37.5°N: Basins S1–S13) and central (38.0°–39.5°N: Basins C01–C06) Japan Trench (Fig. 2, Supplementary Figs S1–S20, Supplementary Table 1). The thickness of the 2011 event deposit varies along the trench axis, being generally thicker in the southern part (e.g., 4.9 (+0.7/−0.1) m in basin S06 and 4.6 (+0.7/−0.1) m in basin S10 (Fig. 2e)), while the event deposit is generally thinner in the central Japan Trench (Fig. 2b). The areal extent of the 2011 event deposit in the southernmost basin (S01; 29.4 (±0.5) km^2^, Supplementary Table 1) is largest among all studied basins. Based on SBP data, we quantify the total areal extent and volume of the 2011 event deposits within the Japan Trench to be at least 125.7 (+4.3/−4.2) km^2^ and 0.187 (+0.045/−0.018) km^3^, respectively (Fig. 4a and Supplementary Table 1). These are considered conservative estimates because SBP data do not image deposits in small depositional basins, such as in the narrow trench axis around 38°N. Such small-scale seafloor roughness is beyond the horizontal resolution of the SBP (~80 m in 8 km water depth). While depositional basins can only be identified by the bathymetry data and be validated by sediment cores, we estimate that additional event-deposit volume missed by SBP data amounts to less than 0.007 km^3^ (Supplementary Table 1).

Limited vertical and horizontal SBP resolution, however, may only partly explain our counter-intuitive findings of limited amount of remobilised surficial sediments deposited in the small trench-basins around 37°30′N–38°40′N (Fig. 2c). This area experienced largest-ever-recorded coseismic horizontal displacement and vertical uplift^11,12,21^ which, intuitively, would be expected to deliver large quantities of remobilised surficial sediment to the trench axis (c.f. ref.^18,25^). However, our findings for this part of the trench around 38°N are consistent with results from several cores that documented the 2011 event deposit to be only 5 to 40 cm in thickness^4,9,15,20^ (Fig. 3). We attribute the limited event deposition in this area to the elevated position of this narrow part of the trench axis compared to its northern and southern counterparts and to the lack of large depositional basins. Gravity-focusing of fine-grained remobilised sediments thus results in an absence of large-scale deposition in the central part of the trench axis while leading to thicker deposits in the respective terminal basins, particularly towards the deeper, southern basins, where our data reveals maximum thickness of the 2011 event deposits of up to 4.9 m.

As an alternative to this sediment-process based explanation we also consider that the mainshock (*M*_*w*_9.0–9.1) of the 2011 earthquake resulted in larger ground accelerations of 1,000–2,000 cm/s^2^ than expected in Ibaraki Prefecture, south of the Tohoku coast^26–28^ (Fig. 4). Such strong ground motions could have also triggered large amounts of surficial sediment remobilisation along the southern continental margin slopes. In addition, the largest aftershock (*M*_*w*_7.9) of the 2011 Tohoku-oki earthquake occurred offshore Ibaraki Prefecture approximately 30 minutes after the mainshock^29^, and could have triggered additional surficial sediment remobilisation in this area. We thus infer that the widespread distribution of the 2011 event deposit over the southern Japan Trench could also be linked to significant remobilisation of surficial sediment offshore Ibaraki Prefecture, triggered by the 2011 mainshock and/or the largest aftershock, and subsequent funnelling and focusing of the muddy density flows through the Nakaminato submarine canyon into the southernmost trench basin (indicated by red arrows in Fig. 4).

## Organic Carbon Export to the Hadal Trench by the 2011 Earthquake

TOC contents of the 2011 event deposit sediments at the southern and central sites are similar, yielding 2.16 (+0.26/−0.63) wt% (core GeoB21804) and 1.57 (±0.18) wt% (cores GeoB16431 and GeoB21823), respectively (Fig. 3; see “Methods”). The TOC values are relatively constant throughout the 2011 event deposit but are generally lower than that of the uppermost few centimetres of the 2011 event deposits measured over the Tohoku continental shelf (1.2–4.5 wt%^16,30^). However, higher TOC contents of sediments from the southern trench (core GeoB21804) than those from the central trench (cores GeoB16431 and GeoB21823) may reflect the aforementioned hypothesis of massive delivery of remobilised surficial seafloor sediment into the southern trench via the Nakaminato submarine canyon.

The TOC data and mapped volume of the 2011 event deposit (see “The 2011 Event Deposit in the Hadal Trench”) allow constraints to be placed the total mass of OC within the 2011 event deposits over the entire studied Japan Trench (see “Methods”). Our results indicate that at least 1.73 (+0.96/−0.72) Tg (10^12^ g) of surficial seafloor sediment OC was remobilised from a large swath of the margin and delivered to the trench by a single tectonic event (i.e., the 2011 Tohoku-oki earthquake and its aftershock sequence). Historical records (e.g., AD 1454 Kyotoku and AD 869 Jogan earthquakes) and turbidites and tsunami deposits during the last 4,000 years suggest that such giant earthquakes occur at an interval of 500–900 years^31,32^. Similar to the 2011 event deposits, these older event deposits also contain relatively young remobilised sediments ascertained by only a few thousand years of offset between background and event deposits^9^, suggesting the occurrence of comparable remobilisation and deposition processes. Thus, event-deposits in trench fill-basins of the Japan Trench act as an OC sink during interseismic periods. Given that sediment cores suggest accumulation of potentially even thicker event deposits in the central Japan Trench following historic earthquakes^9,17^, we estimate that, on average, on the order of 10^0^–10^1^ Tg OC remobilised from surficial slope seafloor sediments is supplied to the trench per giant seismic event. Since interseismic sedimentation rates and carbon fluxes are lower by about two orders of magnitude^9,17^, the mechanism of megathrust earthquake-driven carbon supply to the hadal trench described here accounts for the majority of OC burial and, through eventual underthrusting, carbon sequestration on geological time-scales.

## Relevance to Carbon Cycle in the Deep Sea

The fate of organic matter in the deep ocean is a key factor for understanding of the global carbon cycle^7,33^. Global estimates for the amount and accumulation rate of OC in surface marine sediments are 150 Pg (10^15^ g)^33^ and 170 Tg C per year^34^, respectively. Worldwide fluvial delivery of particulate and dissolved OC from the land to the ocean is estimated to be as high as 0.2–0.5 Pg C per year^35–37^. The Himalayan erosional system alone delivers ~4 Tg C per year of the relatively recent terrestrial OC to the Bengal fan via the Ganges-Brahmaputra rivers systems^38^. However, OC fluxes to the majority of the seafloor are small, averaging ~1 gCm^−2^yr^−1^ (gram of carbon per m^2^ per year)^39^. The major fraction of OC delivered from the continents to the ocean and produced in the ocean that survives remineralisation is buried on the continental margins, with <5% accumulating in abyssal ocean settings^33,34^. Processes that transfer carbon from the margin to the abyssal ocean may therefore exert a strong influence on deep ocean carbon budgets and associated biogeochemical cycles. On the Hikurangi margin, 7 Tg C was transferred by large-scale sediment transport of ~0.9 km^3^ through submarine canyons to the deep ocean following the 2016 Kaikōura earthquake, whose recurrence interval is estimated to be ~150 years^40^. In comparison to these events and processes, the total mass of OC delivered to the Japan Trench (>1 Tg C) by a single tectonic event (here reconstructed for the 2011 Tohoku-oki earthquake and its aftershock sequences) represents an important contribution to marine sediments of hadal zones that may have broader-scale significance.

Notably, the OC concentration per unit area within the 2011 event deposit (4.1 (+3.1/−2.2) to 31.7 (+17.5/−13.8) kgCm^−2^; ave., 12 kgCm^−2^) is an order of magnitude higher than the global average value of OC buried in surface marine sediments (0.4 kgCm^−2^; cf.^33^). The hadal trenches previously studied, including Izu-Bonin (Ogasawara), Mariana, New Britain, Tonga, and Peru-Chile Trenches, have also been recognised as potential depocentres for sediments enriched in organic material (surface sediment OC concentrations range between 0.7–9.2 kg Cm^−2^ (Supplementary Table 2; refs^5,41–43^). However, our newly reported estimates on OC contents of Japan Trench sediments related to the most recent earthquake-triggered remobilisation event rank up to a factor of 2 higher in comparison with corresponding data from other hadal trenches.

It remains to be determined whether the remobilized fresh OC delivered following tectonic events to hadal-trench systems is reactive and subject to microbial degradation^44^ and benthic recycling, or whether it is subducted to contribute to the deep carbon cycle, possibly eventually influencing CO_2_ degassing through the proximal volcanic activity^8^. Nevertheless, our new high-resolution hydroacoustic measurements coupled with sedimentological and geochemical data allow a first-ever trench-wide quantification of megathrust earthquake-induced sediment and OC translocation, and virtually instantaneous supply of >1 Tg OC to the hadal zone. These fluxes are comparable with those described for other Earth system processes, implying potential global significance. Taken in the context of estimates of carbon burial in other trench systems, our findings highlight the importance of tectonic events for carbon cycling in hadal trenches of plate subduction margins.

## Methods

### Bathymetric data

Bathymetric data were acquired by a 12 kHz frequency KONGSBERG EM122 system equipped on *R/V Sonne* during SO251-1 cruise in October 2016 (ref.^45^), which has 432 beams with a transducer of 0.5 (transmission) by 1 (receiving) degrees, generating a footprint of 60–70 meters along track by 130–140 meters across-track in the trench axis, and has a dual swath (multiping) function. Such small transducer configuration, which equals small footprints, together with higher beam counts and dual ping system makes spatial resolution and signal-to-noise ratio of the bathymetric data resulting from this latest state-of-the-art acquisition system uniquely capable of identifying even small depositional basins within the structurally complex and deep-water trench-floor system (Figs 1, 2, Supplementary Figs 1–12).

### Subbottom profilers (SBPs)

SBP data were obtained during six research cruises with two different acquisition systems: SO251-1 (October 2016) and SO219A-2 cruises (March–April 2012) by *R/V Sonne* equipped with ATLAS PARASOUND P70 echosounder and KS-16-14 (September 2016), KS-15-16 (November 2015), KS-15-3 (May 2015), and KS-14-16 cruises (September 2014) by *R/V Shinsei-maru* with KONGSBERG TOPAS PS 18. The PARASOUND echosounder emits two high frequencies of 18 kHz and 22 kHz, and non-linear interference of the high frequencies produces a secondary frequency of about 4 kHz. The TOPAS system uses a primary frequency of 15–21 kHz, and a secondary frequency of 0.5–6.0 kHz. Frequency filtering was done through low-pass bandpass at 6 kHz for PARASOUND data^45^ and high- and low-pass filters at 2 and 7 kHz for TOPAS data. The SBP taken by PARASOUND records mostly at 30–70 m per shot point (SP), while that taken by TOPAS system at 10–40 m per SP. The theoretical vertical resolutions of all SBP data are 10–20 cm, which enabled identifying the decimetre to meter scale event deposit layers (Fig. 1, Supplementary Figs 1–12). For better visual continuity of the reflections, the trace envelope of the SBP data is shown. We postprocessed the data for cases of noisy SBP data due to adverse weather conditions or interference with the ship’s multibeam pings (see Supplementary Information).

### Interpretation of the 2011 event deposit

Following standard seismic-stratigraphic interpretation methods we pick the top and bottom horizon of the interpreted 2011 event deposit (see “The 2011 Event Deposit in the Hadal Trench” for event-deposit interpretation; Fig. 1, Supplementary Figs 1–12). We then estimate areal extent and volume of 2011 event deposits in individual trench-fill basins, each guided by integrating SBP and multibeam bathymetry data (see Supplementary Information). Uncertainties in areal extent and volume of the 2011 event deposit at a given basin are also estimated (see Supplementary Information), taking into account resolution of bathymetry (±50 m of lateral resolution), and SBP data (±10 cm vertical resolution; variable thickness upon the choice of internal velocity of event deposit possibly ranging from 1,500 to 1,700 m/s).

### Radionuclide dating, total organic carbon (TOC) content, and dry density measurements of sediment cores

To correlate acoustically-transparent bodies from SBP data to the event deposit triggered by the 2011 earthquake, and to measure carbon content and estimate carbon mass flux to the hadal trench, we use existing core data from cores MR12-E01-PL03 (ref.^17^) and GeoB16431 (ref.^9^), and add new data from the cores GeoB21804 and GeoB21823, retrieved during *R/V Sonne* cruise SO251-1 in 2016 (ref.^45^).

The radioactivity of ^210^Pb (with a half-life of 22.3 yr) from the core GeoB21804 was measured gamma spectrometry at the Rensellaer Polytechnique Institute (Fig. 3 and Supplementary Table 3). The excess ^210^Pb was defined following the method described by Appleby *et al*.^46^.

Total organic carbon (TOC) content (wt%) of the deep-sea trench sediments was quantified by using the Elemental Analyser (EA; Elementar, Germany) at ETH Zürich, with a relative standard deviation (RSD) of 2% (ref.^47^). For this purpose, about 15 mg of freeze-dried and powdered subsamples of the cores GeoB21804, GeoB16431, and GeoB21823 were weighed into pre-combusted Ag capsules. The Ag capsules containing the sediment were placed on a ceramic plate in a desiccator and fumigated for 72 h at 60 °C with ~30 ml of concentrated HCl (37%, metal-trace purity), placed in a pre-combusted petri dish at the bottom of the desiccator. The acidified subsamples were neutralised with ~20 g NaOH pellets for another 72 h at 60 °C. TOC contents are 2.16 (+0.26/−0.63) wt% and 1.57 (±0.18) wt% in the southern (from GeoB21804 core) and central Japan Trench (GeoB16431 and GeoB21823 cores), respectively (Fig. 3).

Dry density of the 2011 event deposit was calculated from the ratio of dry mass and total volume of the sample taken from the cores GeoB21804 and GeoB16431. The dry mass was corrected for the mass of salt that remains within the pore space after drying in an oven at 100 °C for 24 hours. The total volume consists of the sum of the volume of water and the volume of grains. The volume of water was derived from the water content determined following DIN EN ISO 17892-1. The volume of grains was determined with a gas pycnometer (Pentapyc 5200e, Quantachrome Instruments), following DIN 66137-2 (Determination of solid state density–Part 2: Gaspycnometry, 2004). Dry densities of the 2011 event deposit from the cores GeoB21804 and GeoB21823 were *ρ*_*d,S*_ = 330–430 kg/m^3^ and *ρ*_*d,C*_ = 790–950 kg/m^3^, respectively. The difference in dry density form the southern and central area may be explained by the fact that compaction of southern thick event deposits supplied through the proximal canyon has not been completed, as also indicated by very low undrained share strength values for the uppermost few meters of the cores^45^.

### Mass of organic carbon in the 2011 event deposit

Masses of organic carbon in the southern (36.0°–37.5°N) and central Japan Trench (38.0°–39.5°N) are calculated using the measured TOC contents and dry densities from core GeoB21804 and those from the cores GeoB16431 and GeoB21823, respectively (see the previous Method subsection “Radionuclide dating, total organic carbon (TOC) content, and dry density measurements of sediment cores”). Mass of organic carbon *mOC*_*S,i*_ (kg) at a basin *i* in the southern Japan Trench (*i* = S01, S02, …, S13; Supplementary Table 1) is produced by:

$$mO{C}_{S,i}\,({\rm{k}}{\rm{g}})={{\rm{T}}{\rm{O}}{\rm{C}}}_{{\rm{S}}}\times {\rho }_{d,S}\,({\rm{k}}{\rm{g}}/{{\rm{m}}}^{3})\times {V}_{S,i}\,({{\rm{m}}}^{3}),$$

where TOC_S_ (wt%) is TOC content measured from the core GeoB21804, *ρ*_*d,S*_ (kg/m^3^) is the dry density measured from the core GeoB21804 (see the previous Method subsection “Radionuclide dating, total organic carbon (TOC) content, and dry density measurements of sediment cores”), and *V*_*S,i*_ (m^3^) is volume of the 2011 event deposit at the basin *i* in the southern Japan Trench obtained by bathymetry and SBP data (Supplementary Table 1). Similarly, mass of organic carbon *mOC*_*C,i*_ (kg) at a basin *i* in the central Japan Trench (*i* = C01, C02, …; Supplementary Table 1) is produced by:

$$mO{C}_{C,i}\,({\rm{k}}{\rm{g}})={{\rm{T}}{\rm{O}}{\rm{C}}}_{{\rm{C}}}\times {\rho }_{d,C}\,({\rm{k}}{\rm{g}}/{{\rm{m}}}^{3})\times {V}_{C,i}\,({{\rm{m}}}^{3}),$$

where TOC_C_ (wt%) is TOC content measured from the cores GeoB16431 and GeoB21823, *ρ*_*d,C*_ (kg/m^3^) is the dry density measured from the core GeoB21823 (see the previous Method subsection “Radionuclide dating, total organic carbon (TOC) content, and dry density measurements of sediment cores”), and *V*_*C,i*_ (m^3^) is volume of the 2011 event deposit at the basin *i* in the central Japan Trench obtained by bathymetry and SBP data (Supplementary Table 1). The organic carbon masses *mOC*_*S,i*_ and *mOC*_*C,i*_ are computed taking into account the uncertainty in volume determination (Supplementary Table 1).

## Supplementary information

Supplementary Information

## Data Availability

Bathymetric data used in the paper are available at Bundesamt für Seeschifffahrt und Hydrographie (https://www.bsh.de/DE/DATEN/Ozeanographisches_Datenzentrum/Vermessungsdaten/Nordpazifischer_Ozean/nordpazifik_node.html) and JAMSTEC-DARWIN database (http://www.godac.jamstec.go.jp/darwin/e). The other data that support the findings of this study are available from the corresponding author (A.K.) upon reasonable request.
