# Supplementary material for: Megathrust earthquake drives drastic organic carbon supply to the hadal trench
Source: Sci Rep. 2019 Feb 7;9:1553. doi: 10.1038/s41598-019-38834-x (PMC6367409; doi:10.1038/s41598-019-38834-x)
Supplement: Supplementary file 1 — Supplementary Information [file 41598_2019_38834_MOESM1_ESM.docx]

SUPPLEMENTARY INFORMATION for

**Megathrust earthquake drives drastic organic carbon supply to the hadal trench**

A. Kioka, T. Schwestermann, J. Moernaut, K. Ikehara, T. Kanamatsu, C. M. McHugh, C. dos Santos Ferreira, G. Wiemer, N. Haghipour, A. J. Kopf, T. I. Eglinton & M. Strasser

This SUPPLEMENTARY INFORMATION includes:

- Supplementary Figures 1 to 20,
- Supplementary Tables 1 to 3.

**1. Introduction for SUPPLEMENTARY INFORMATION**

Section 2 addresses the processing of the studied subbottom profiler (SBP) data for cases of noisy SBP data. Section 3 provides descriptions regarding delineation of trench-fill basins, for which SBP data resolves acoustically-transparent event deposits on the seafloor (inferred to relate to the 2011 event; Supplementary Fig. 1 – 12), and generation of event-deposit thickness maps within these basins (Supplementary Fig. 13 – 20). Supplementary Table 1 lists thickness, area, and volume of the 2011 event deposit at studied trench-fill basins. Supplementary Table 2 summarizes total organic carbon (TOC) contents in the surface sediment layers of the hadal trenches worldwide. Supplementary Table 3 shows the xs210Pb data from the newly reported core.

2. Processing of noise attenuation of SBP data

Studied SBP data acquired by both Parasound (R/V Sonne) and Topas system (R/V Shinsei-Maru) often contain noisy traces that are caused by either interference of the ship’s multibeam system or bad weather conditions (i.e., swell). Unlike the synthetic datasets, the commonly used signal-to-noise ratio measurement cannot be applied for the observed acoustic datasets. Therefore, for the moderately noisy SBP data, we improved the data quality to ensure identification of acoustically transparent bodies, by removing bad traces and interpolation of the resultant irregularly-populated trace data. Interpolation of the killed traces in a given seismic data was solved as a sparse inverse problem using the projection-onto-convex sets algorithm (Abma & Kabir, 2006). We used the method of soft thresholding by the iterative shrinkage-thresholding algorithm (Daubechies et al., 2004) with sufficiently high thresholding level, and applied 1,000 iterations to assure the convergence. For the noisier SBP data, we skipped the interpolation processing for several traces because it is sensitive to neighbouring shot traces, but alternatively processed attenuation of possible noises using local signal-and-noise orthogonalisation (Chen & Fomel, 2015). SBP data were processed using CWP/SU (Cohen and Stockwell, 2015) and Madagascar open-source platform (Fomel et al., 2013).

3. Delineation of basins and calculation of thickness and volume of the 2011 event deposit

Areal extent and volume of the 2011 event deposit in a given trench-fill basin were obtained using bathymetry data from SO251-1 cruise and SBP data from SO251-1, SO219A-2, KS-16-14, KS-15-16, KS-15-3, and KS-14-16 cruises (see Supplementary Table 1 for summarized data): First, acoustically transparent bodies with ponding geometries occurring immediately below the seafloor reflector (i.e. seafloor coincides with top of mappable unit inferred to be 2011 event deposit) were identified in SBPs (yellow layers in Supplementary Fig. 1–12). This determined lateral pinch-out locations of the 2011 event deposit (e.g., the locations are guided by A/A’, B/B’, and C/C’ in Supplementary Fig. 1–12) and produced two-way travel time differences between top and base of the 2011 event deposit (Supplementary Fig. 13a–20a) along the studied SBP lines at a given basin. Second, the areal extent of the respective basin documenting the 2011 event deposit was delineated (red closed curves in Supplementary Fig. 1a–12a) in light of 2-m contours of high-resolution bathymetric data acquired by *R/V Sonne* in October 2016, and constrained by the observed pinch-out locations of the 2011 event deposit in SBPs. The uncertainty for delineating the areal extent of the 2011-event deposits is ±50 m in X and Y, taking into account the sampling interval of the bathymetric data we used is 100 m. Third, an isochron (sediment thickness shown in two-way travel time) map at a given extended basin was made from the two-way travel time of the 2011 event deposit (Supplementary Fig. 13b–S20b). Two gridding algorithms available from IHS Kingdom 2015 software were used for generating isochron maps. For the basin that has ≥2 SBP lines, we used a “Flex Gridding” algorithm that solves a biharmonic problem with parameters “Midway (0.5)” fitness and “Midway (6)” smoothness. For the basin that has only one SBP line, we used a “Minimum Curvature” algorithm with “Minimum (1)” smoothness. Grid cell sizes were set to 20 m. Fourth, a thickness map of the 2011 event deposit was made using the generated isochron map upon the choice of internal velocity of 1,500 m/s (Fig. 2 in main text). An uncertainty in thickness does not only relate to the vertical resolution of studied SBP data (± ~0.1 m) but also to the velocity that yields +13% when the velocity ranges between 1,500 and 1,700 m/s. Finally, volume of the 2011 event deposit at a given basin was calculated using the generated thickness map. The uncertainty of volume is large when a studied volume is small, as it is propagated from the uncertainties of thickness and basin area (see Supplementary Table 1).

The reliability of estimated areal extent and volume of event deposit at a given basin depends on the number of available SBP lines used for better delineating of the trench-fill basin. This study thus classifies the reliability of the basin data into three ranks A, B, C (Supplementary Table 1), based on the following criteria: reliability A is the trench-fill basin is delineated by ≥3 SBP lines along- and across the basin; reliability B is the trench-fill basin delineated by one along- & more than one across-Basin SBP lines, or, one across- & more than one along-Basin SBP lines; reliability C is the trench-fill basin delineated by only either along- or across-Basin SBP profiles.

**References unique to SUPPLEMENTARY INFORMATION**

Abma, R. & Kabir, N. 3D interpolation of irregular data with a POCS algorithm. *GEOPHYSICS* **71,** E91–E97 (2006).

Chen, Y. & Fomel, S. Random noise attenuation using local signal-and-noise orthogonalization. *GEOPHYSICS* **80,** WD1-WD9 (2015).

Cohen, J. K., & Stockwell, Jr. J. W. CWP/SU: Seismic Un*x Release No. 44: an open source software package for seismic research and processing, Center for Wave Phenomena, Colorado School of Mines (2015).

Daubechies, I., Defrise, M. & De Mol, C. An iterative thresholding algorithm for linear inverse problems with a sparsity constraint. *Commun. Pure Appl. Math.* **57,** 1413–1457 (2004).

Fomel, S., Sava, P., Vlad, I., Liu, Y. & Bashkardin, V. Madagascar: open-source software project for multidimensional data analysis and reproducible computational experiments. *J. Open Res. Softw.* **1,** e8 (2013).


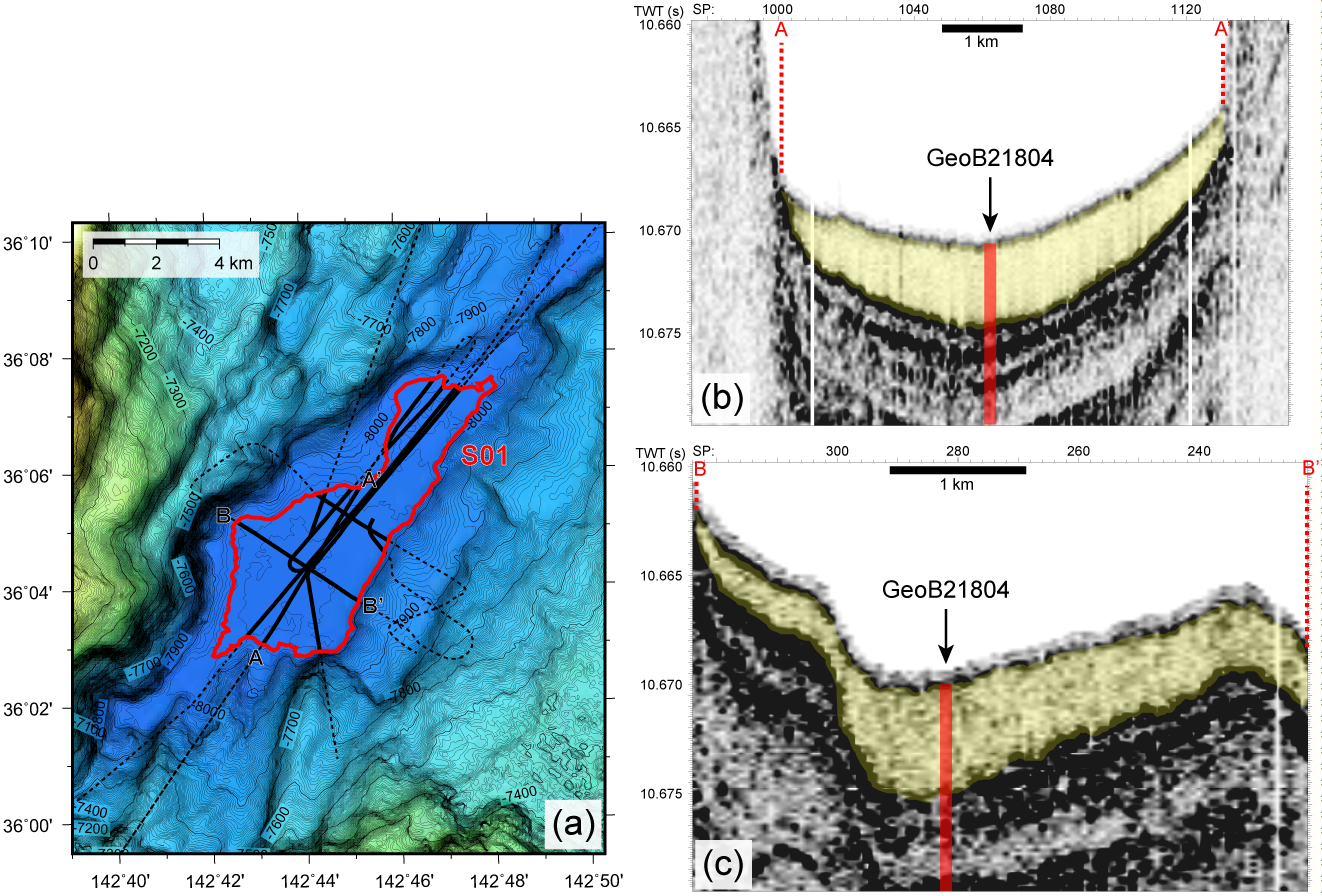


Supplementary Figure 1. (a) Bathymetric map, SBP lines, and delineation of the Basin S01. Contour interval is 5 m. Solid black lines show SBP track lines where the surficial, acoustically transparent body with ponding geometries (i.e., the 2011 event deposits) are identified, while dash lines show SBP track lines without evidence for the event deposits. (b) Example of along-trench SBP image acquired by Parasound system during R/V Sonne SO251-1 cruise. (c) Example of across-trench SBP image acquired by Parasound system during R/V Sonne SO251-1 cruise. Yellow transparent layers highlight the surficial, acoustically-transparent bodies with ponding geometries inferred to be the 2011 event deposits.


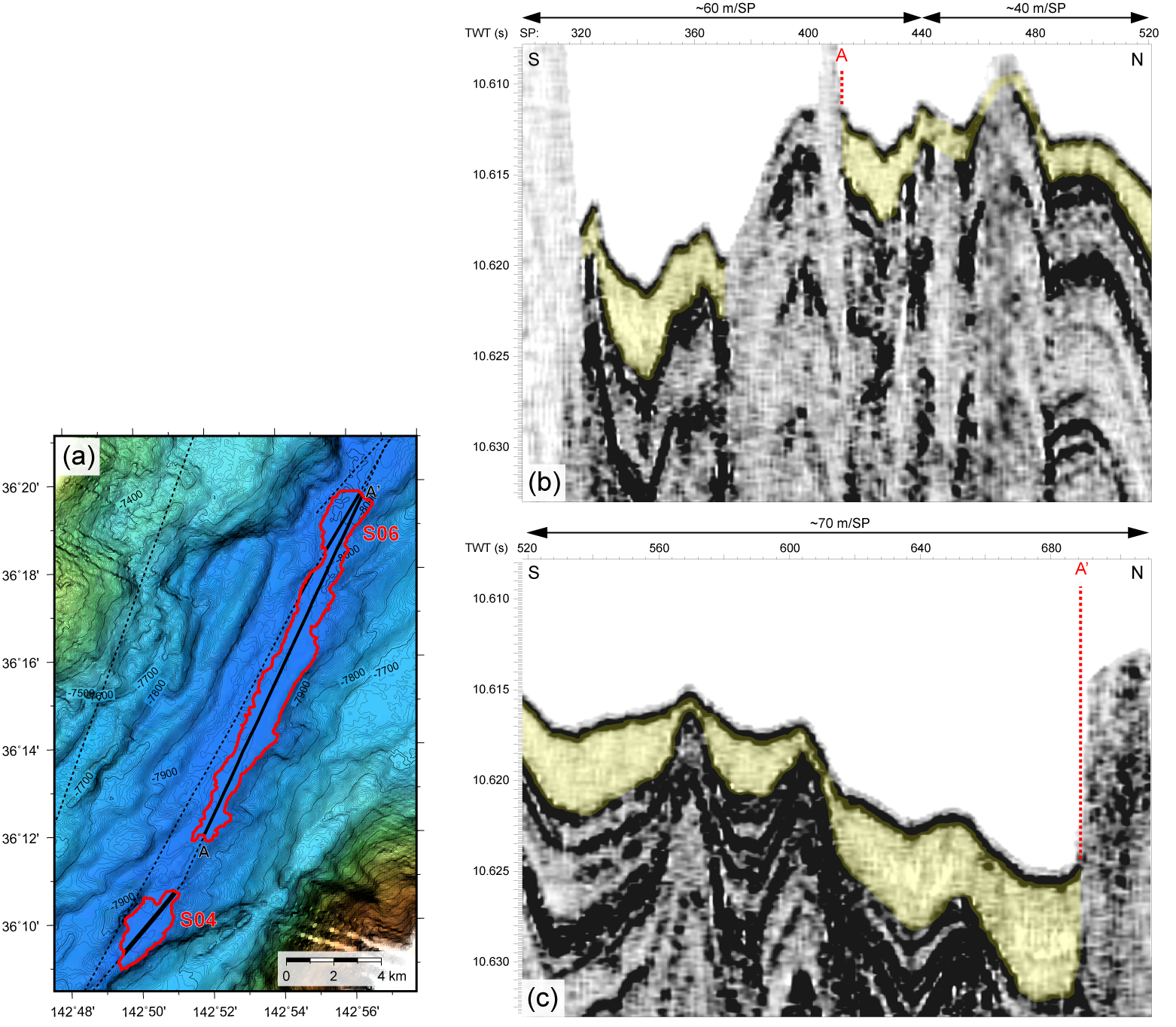


Supplementary Figure 2. (a) Bathymetric map, SBP lines, and delineation of the Basin S06. Contour interval is 5 m. Solid black lines show SBP track lines where the surficial, acoustically transparent body with ponding geometries (i.e., the 2011 event deposits) are identified, while dash lines show SBP track lines without evidence for the event deposits. (b) Example of noise-attenuated SBP image along the trench acquired by Parasound system during R/V Sonne SO251-1 cruise. (c) Example of noise-attenuated SBP image along the trench acquired by Parasound system during R/V Sonne SO251-1 cruise. Yellow transparent layers highlight the surficial, acoustically-transparent bodies with ponding geometries inferred to be the 2011 event deposits.


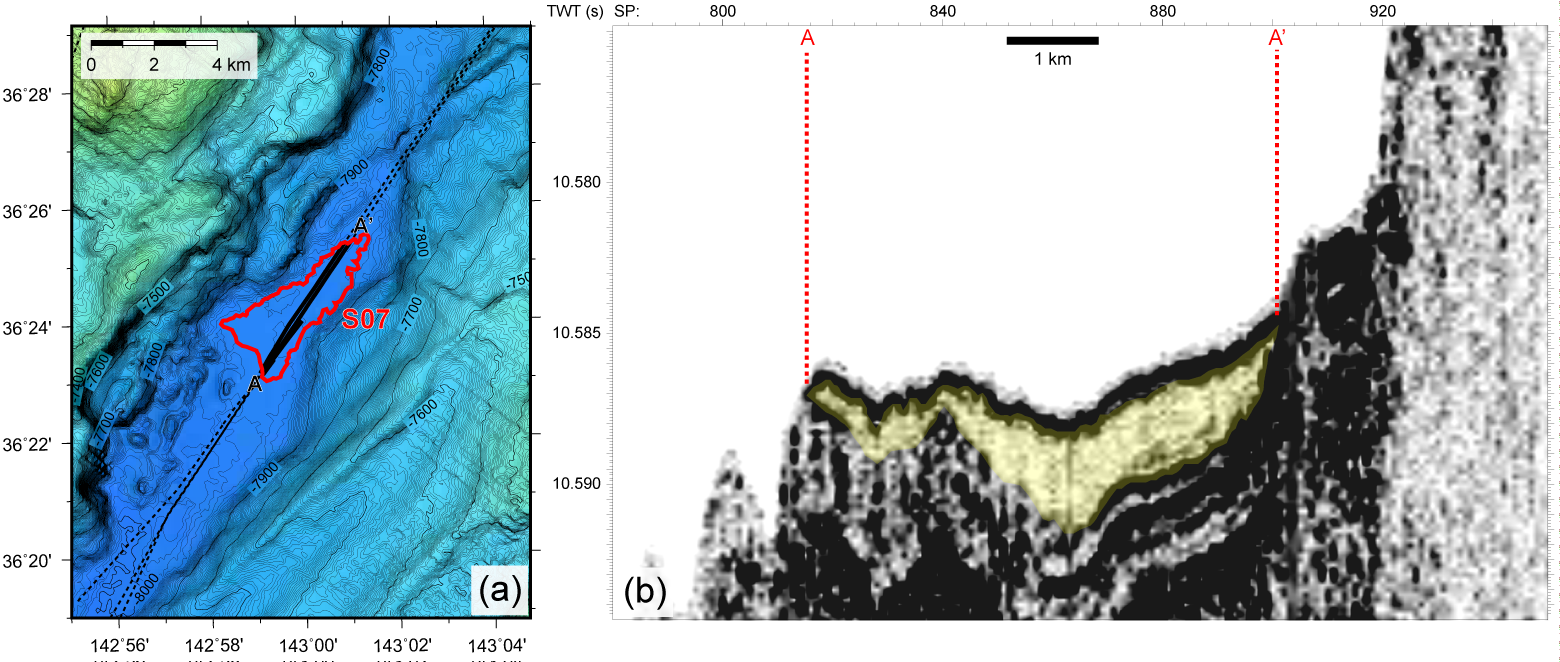


Supplementary Figure 3. (a) Bathymetric map, SBP lines, and delineation of the Basin S07. Contour interval is 5 m. Solid black lines show SBP track lines where the surficial, acoustically transparent body with ponding geometries (i.e., the 2011 event deposits) are identified, while dash lines show SBP track lines without evidence for the event deposits. (b) Example of noise-attenuated SBP image along the trench acquired by Parasound system during R/V Sonne SO251-1 cruise. Yellow transparent layers highlight the surficial, acoustically-transparent bodies with ponding geometries inferred to be the 2011 event deposits.


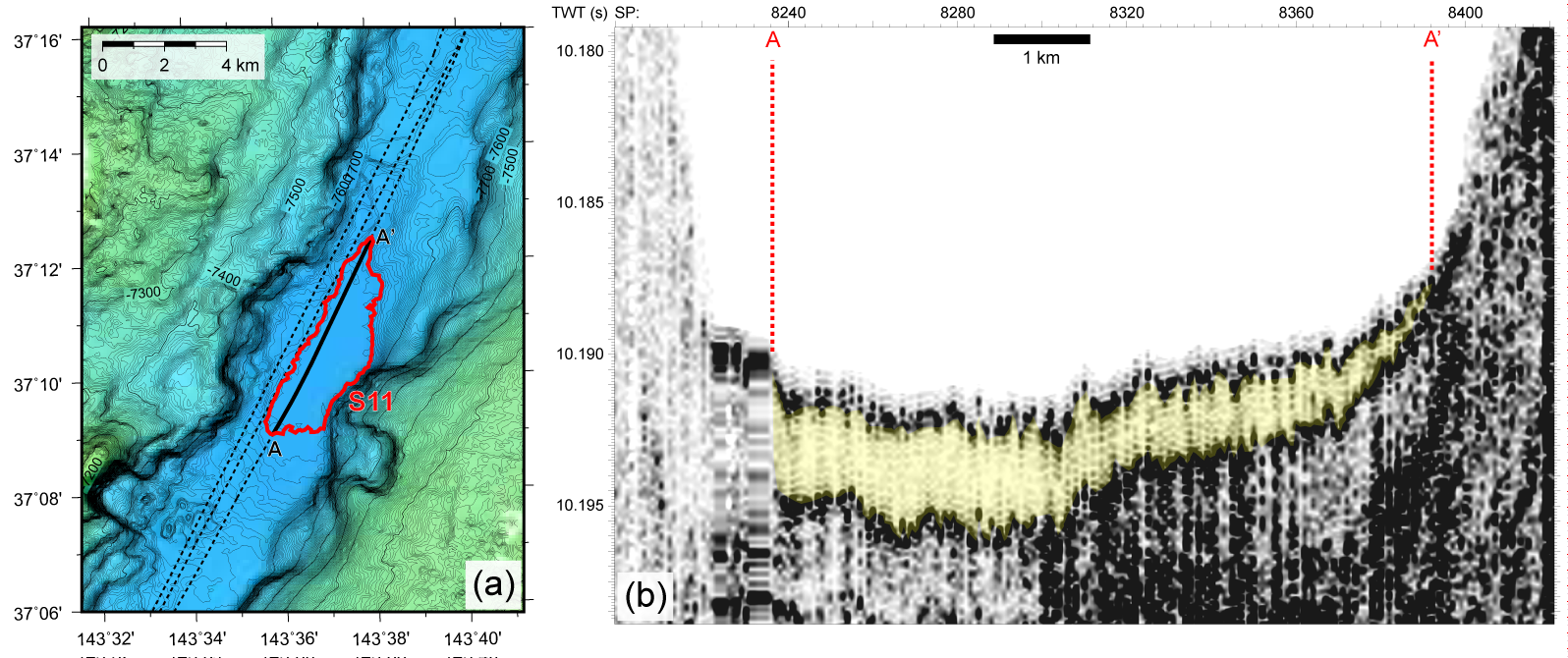


Supplementary Figure 4. (a) Bathymetric map, SBP lines, and delineation of the Basin S11. Contour interval is 5 m. Solid black lines show SBP track lines where the surficial, acoustically transparent body with ponding geometries (i.e., the 2011 event deposits) are identified, while dash lines show SBP track lines without evidence for the event deposits. (b) Noise-attenuated SBP image acquired by TOPAS system during R/V Shinsei-Maru KS-14-16 cruise. Yellow transparent layers highlight the surficial, acoustically-transparent bodies with ponding geometries inferred to be the 2011 event deposits.


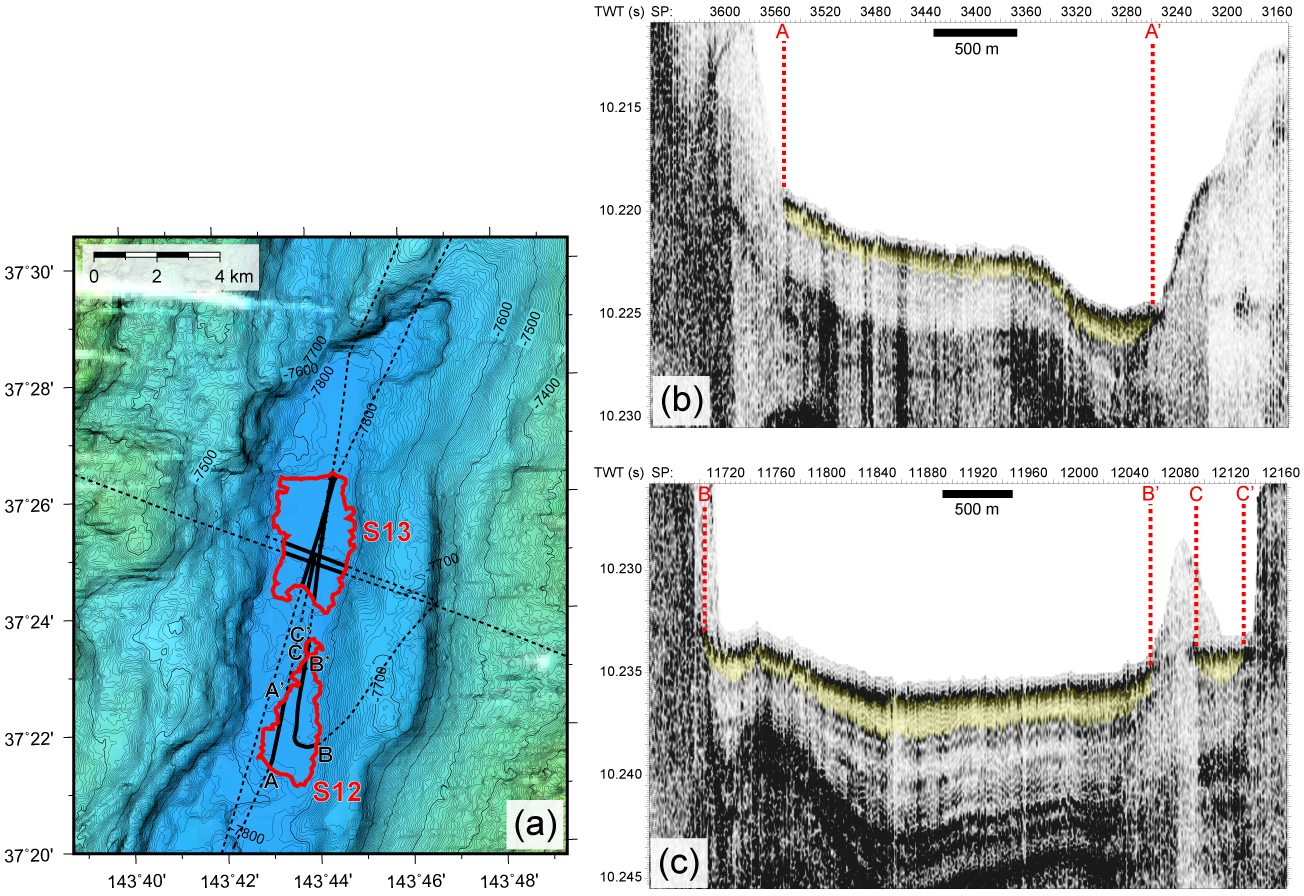


Supplementary Figure 5. (a) Bathymetric map, SBP lines, and delineation of the Basin S12. Contour interval is 5 m. Solid black lines show SBP track lines where the surficial, acoustically transparent body with ponding geometries (i.e., the 2011 event deposits) are identified, while dash lines show SBP track lines without evidence for the event deposits. (b) Along-trench SBP image acquired by TOPAS system during R/V Shinsei-Maru KS-16-14 cruise. (c) Along- and across-trench SBP image acquired by TOPAS system during R/V Shinsei-Maru KS-15-3 cruise. Yellow transparent layers highlight the surficial, acoustically-transparent bodies with ponding geometries inferred to be the 2011 event deposits.


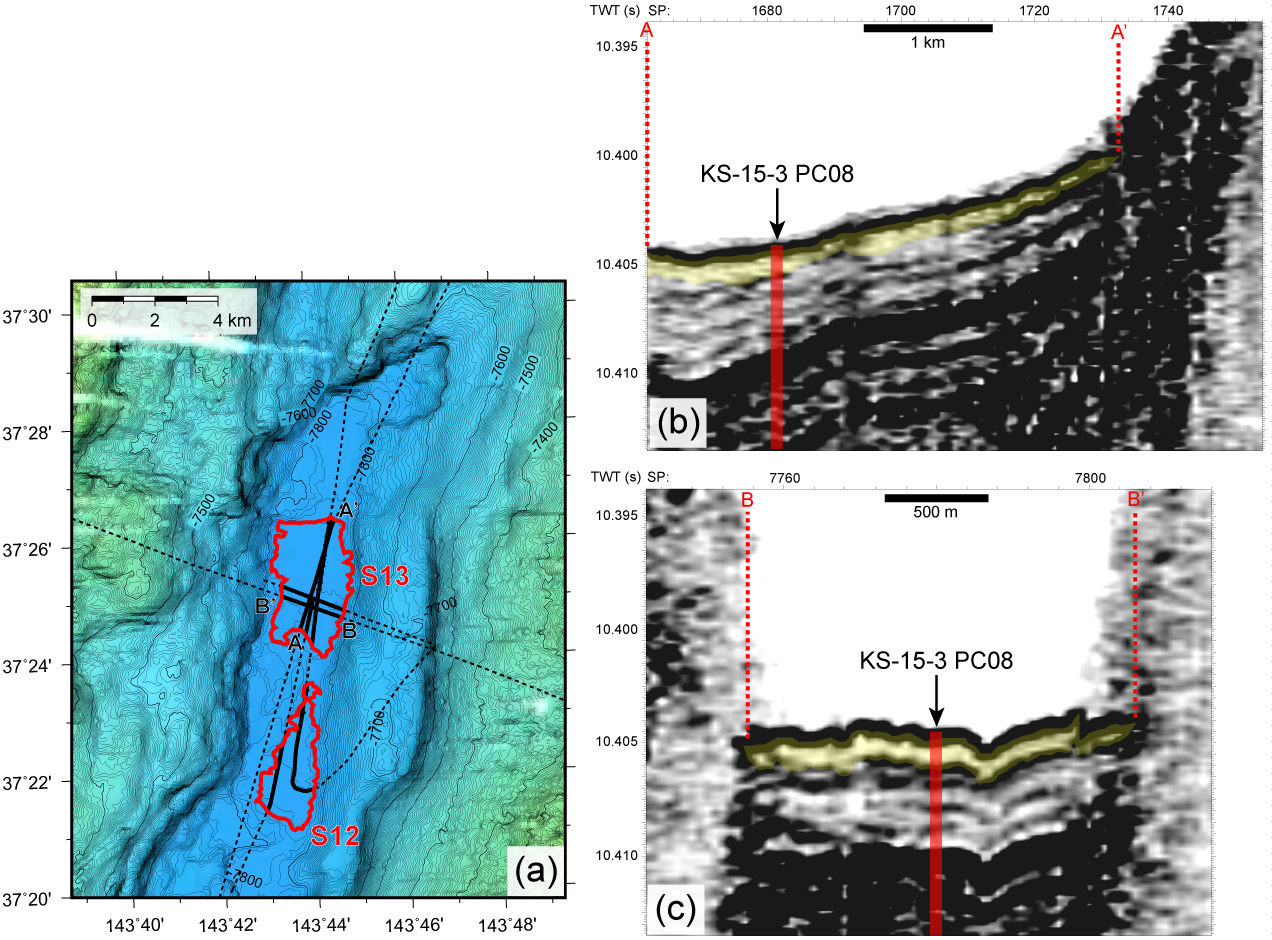


Supplementary Figure 6. (a) Bathymetric map, SBP lines, and delineation of the Basin S13. Contour interval is 5 m. Solid black lines show SBP track lines where the surficial, acoustically transparent body with ponding geometries (i.e., the 2011 event deposits) are identified, while dash lines show SBP track lines without evidence for the event deposits. (b) Example of noise-attenuated SBP image along the trench acquired by Parasound system during R/V Sonne SO251-1 cruise. (c) Example of noise-attenuated SBP image across the trench acquired by Parasound system during SO219A-2 cruise. Yellow transparent layers highlight the surficial, acoustically-transparent bodies with ponding geometries inferred to be the 2011 event deposits.


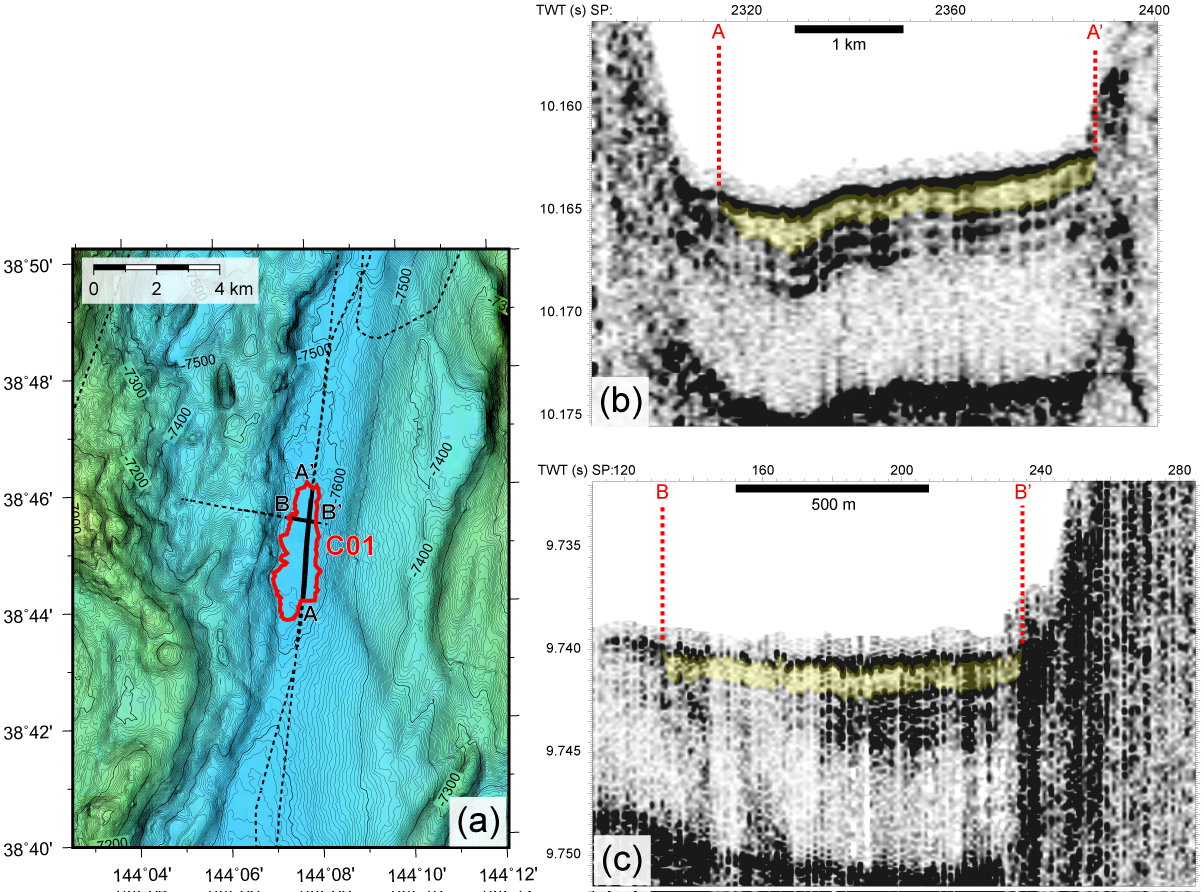


Supplementary Figure 7. (a) Bathymetric map, SBP lines, and delineation of the Basin C01. Contour interval is 5 m. Solid black lines show SBP track lines where the surficial, acoustically transparent body with ponding geometries (i.e., the 2011 event deposits) are identified, while dash lines show SBP track lines without evidence for the event deposits. (b) Noise-attenuated SBP image along the trench acquired by Parasound system during R/V Sonne SO251-1 cruise. (c) Noise-attenuated SBP image across the trench acquired by TOPAS system during R/V Shinsei-Maru KS-15-16 cruise. Yellow transparent layers highlight the surficial, acoustically-transparent bodies with ponding geometries inferred to be the 2011 event deposits.


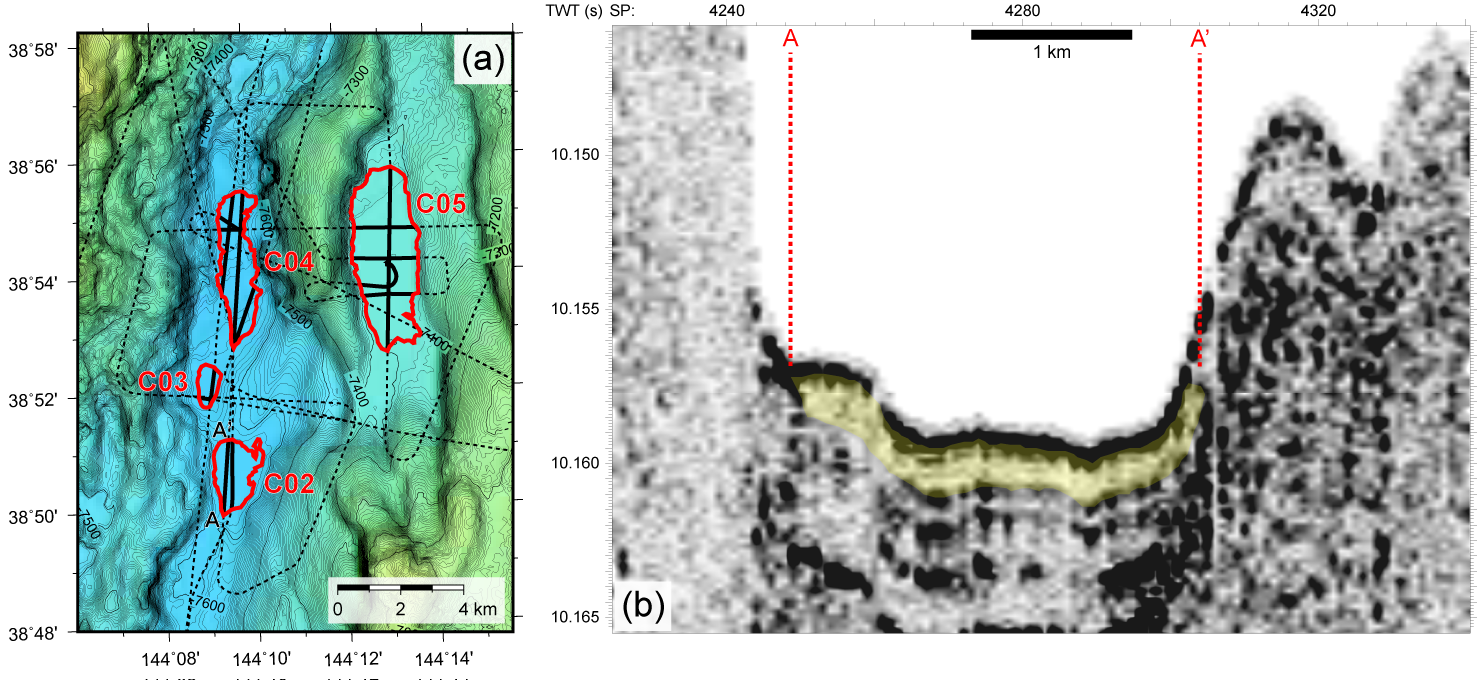


Supplementary Figure 8. (a) Bathymetric map, SBP lines, and delineation of the Basin C02. Contour interval is 5 m. Solid black lines show SBP track lines where the surficial, acoustically transparent body with ponding geometries (i.e., the 2011 event deposits) are identified, while dash lines show SBP track lines without evidence for the event deposits. (b) Example of noise-attenuated SBP image along the trench acquired by Parasound system during R/V Sonne SO251-1 cruise. Yellow transparent layers highlight the surficial, acoustically-transparent bodies with ponding geometries inferred to be the 2011 event deposits.


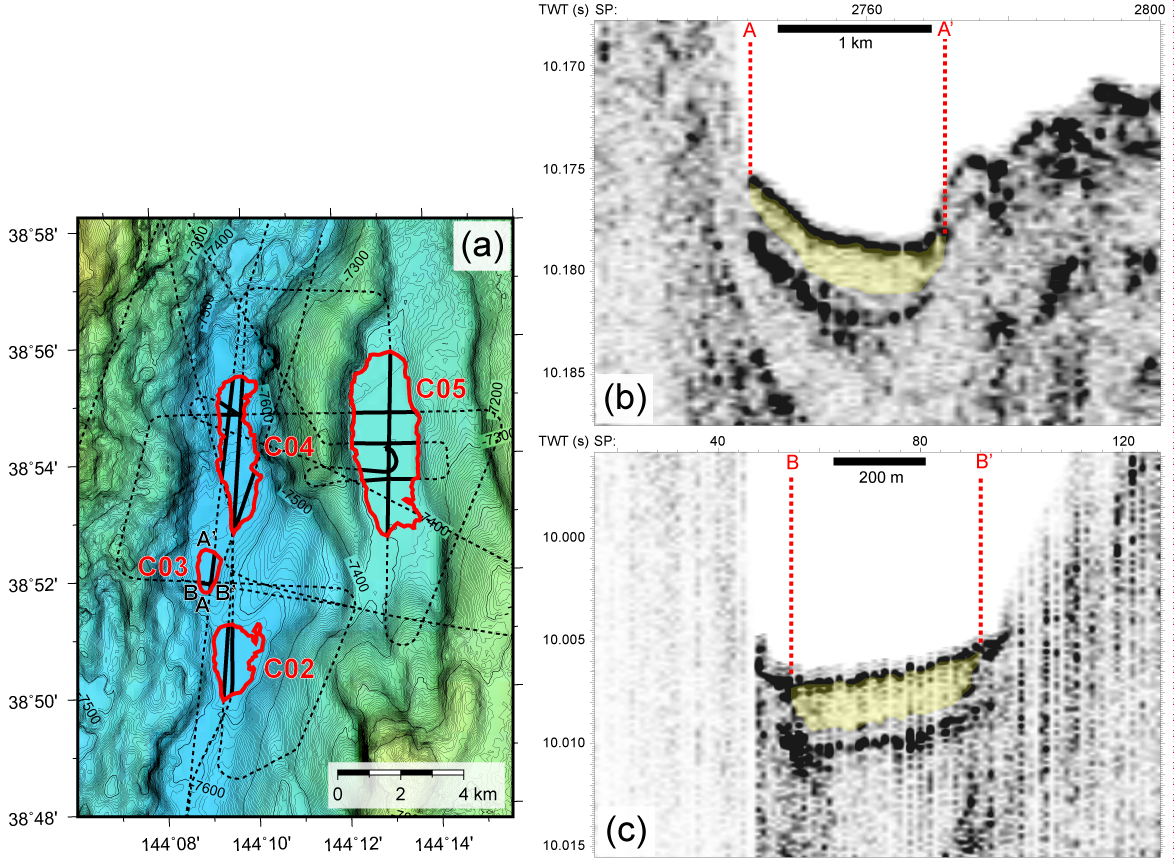


Supplementary Figure 9. (a) Bathymetric map, SBP lines, and delineation of the Basin C03. Contour interval is 5 m. Solid black lines show SBP track lines where the surficial, acoustically transparent body with ponding geometries (i.e., the 2011 event deposits) are identified, while dash lines show SBP track lines without evidence for the event deposits. (b) Example of noise-attenuated SBP image along the trench acquired by Parasound system during R/V Sonne SO251-1 cruise. (c) Example of SBP image across the trench acquired by TOPAS system during R/V Shinsei-Maru KS-15-16 cruise. Yellow transparent layers highlight the surficial, acoustically-transparent bodies with ponding geometries inferred to be the 2011 event deposits.


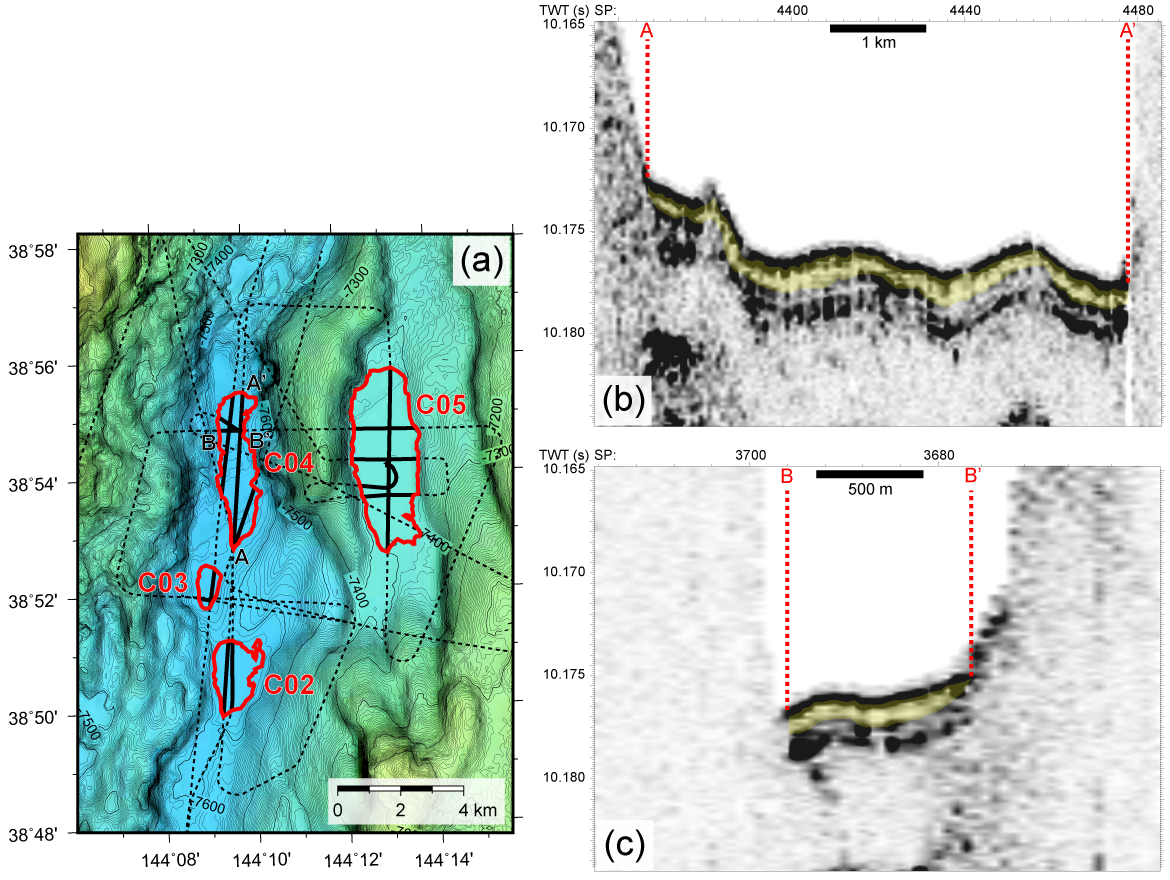


Supplementary Figure 10. (a) Bathymetric map, SBP lines, and delineation of the Basin C04. Contour interval is 5 m. Solid black lines show SBP track lines where the surficial, acoustically transparent body with ponding geometries (i.e., the 2011 event deposits) are identified, while dash lines show SBP track lines without evidence for the event deposits. (b) Example of noise-attenuated SBP image along the trench acquired by Parasound system during R/V Sonne SO251-1 cruise. (c) Example of noise-attenuated SBP image across the trench acquired by Parasound system during R/V Sonne SO251-1 cruise. Yellow transparent layers highlight the surficial, acoustically-transparent bodies with ponding geometries inferred to be the 2011 event deposits.


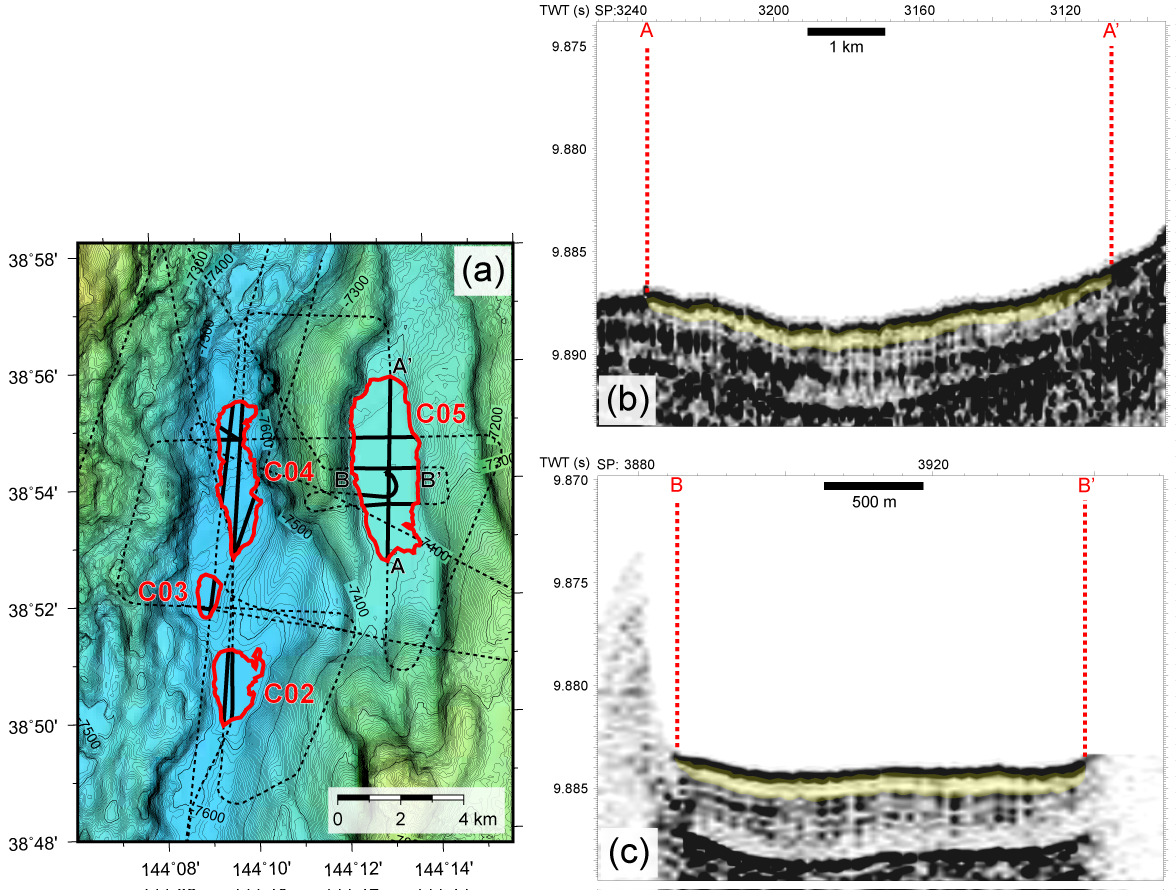


Supplementary Figure 11. (a) Bathymetric map, SBP lines, and delineation of the Basin C05. Contour interval is 5 m. Solid black lines show SBP track lines where the surficial, acoustically transparent body with ponding geometries (i.e., the 2011 event deposits) are identified, while dash lines show SBP track lines without evidence for the event deposits. (b) Example of noise-attenuated SBP image along the trench acquired by Parasound system during R/V Sonne SO251-1 cruise. (c) Example of SBP image across the trench acquired by Parasound system during R/V Sonne SO251-1 cruise. Yellow transparent layers highlight the surficial, acoustically-transparent bodies with ponding geometries inferred to be the 2011 event deposits.


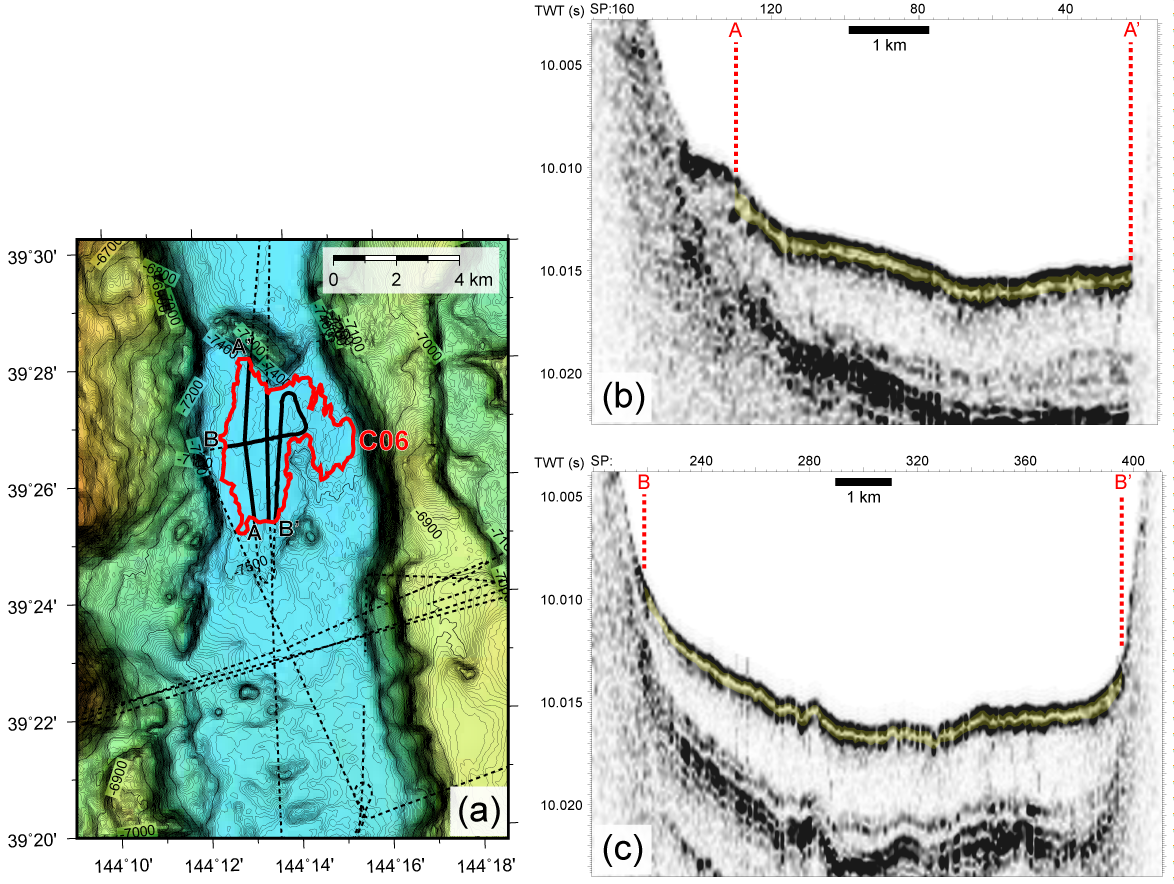


Supplementary Figure 12. (a) Bathymetric map, SBP lines, and delineation of the Basin C06. Contour interval is 5 m. Solid black lines show SBP track lines where the surficial, acoustically transparent body with ponding geometries (i.e., the 2011 event deposits) are identified, while dash lines show SBP track lines without evidence for the event deposits. (b) Example of noise-attenuated SBP image along the trench acquired by Parasound system during R/V Sonne SO251-1 cruise. (c) Example of SBP image along and across the trench acquired by Parasound system during R/V Sonne SO251-1 cruise. Yellow transparent layers highlight the surficial, acoustically-transparent bodies with ponding geometries inferred to be the 2011 event deposits.


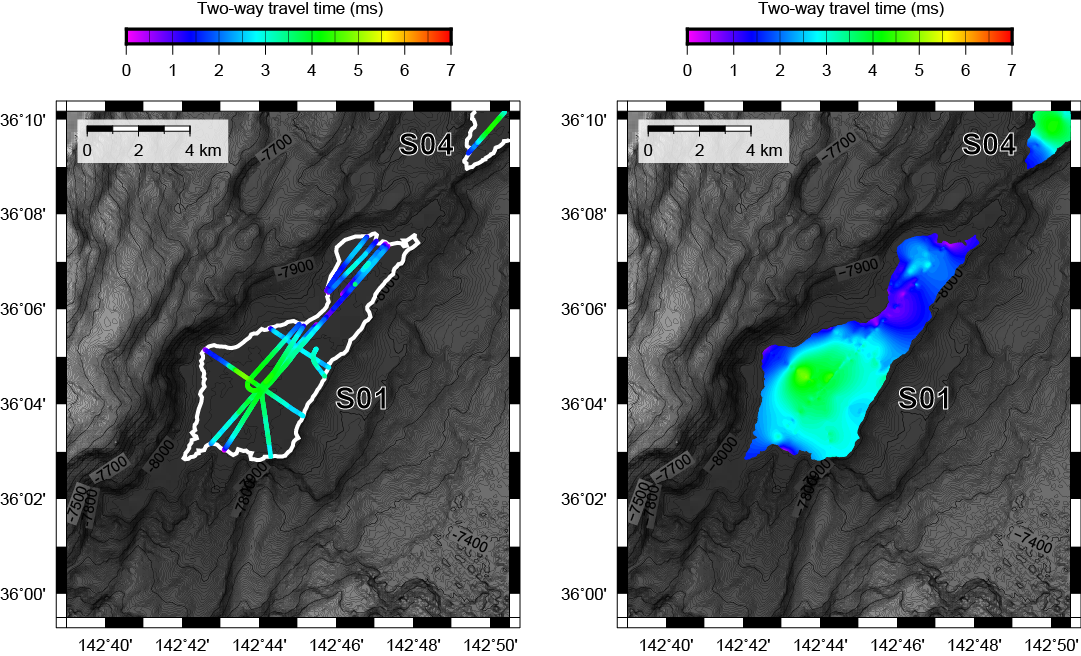


Supplementary Figure 13. (Left) Two-way travel time (TWT) difference between top and base of the 2011 event deposit along the studied SBP lines at the Basin S01 (see Supplementary Figure 1). (Right) TWT-Isopach map of the 2011 event deposit over the entire Basin S01.


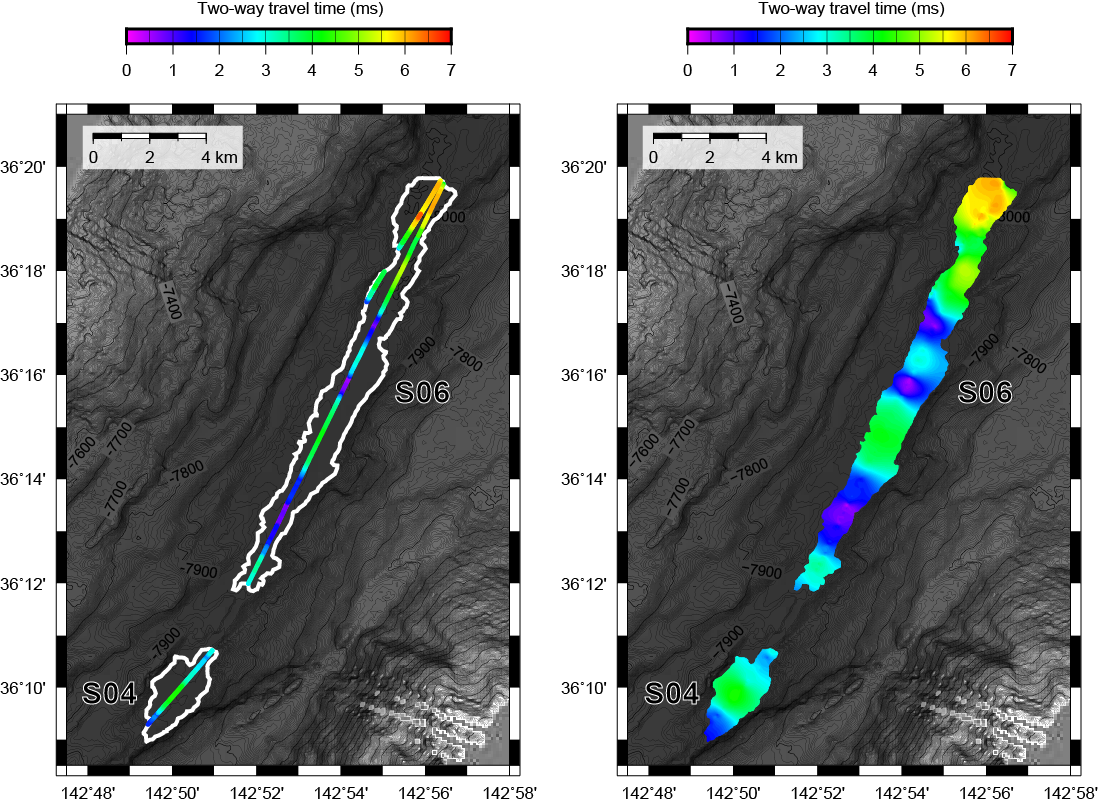


Supplementary Figure 14. (Left) Two-way travel time (TWT) difference between top and base of the 2011 event deposit along the studied SBP lines at the Basin S06 (see Supplementary Figure 2). (Right) TWT-Isopach map of the 2011 event deposit over the entire Basin S06.


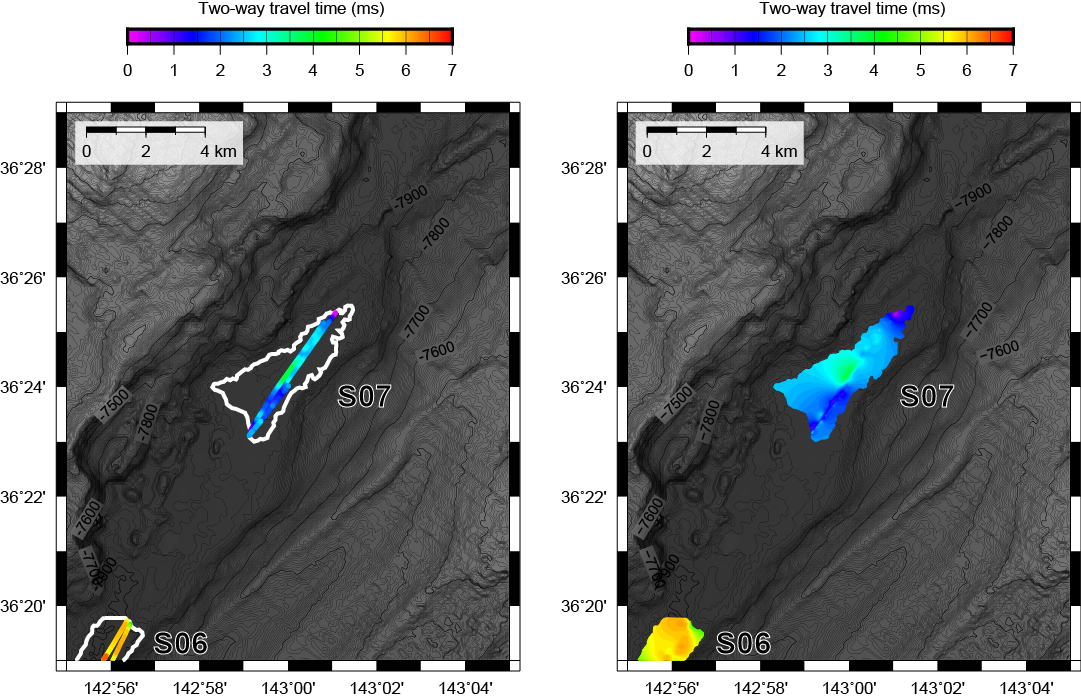


Supplementary Figure 15. (Left) Two-way travel time (TWT) difference between top and base of the 2011 event deposit along the studied SBP lines at the Basin S07 (see Supplementary Figure 3). (Right) TWT-Isopach map of the 2011 event deposit over the entire Basin S07.


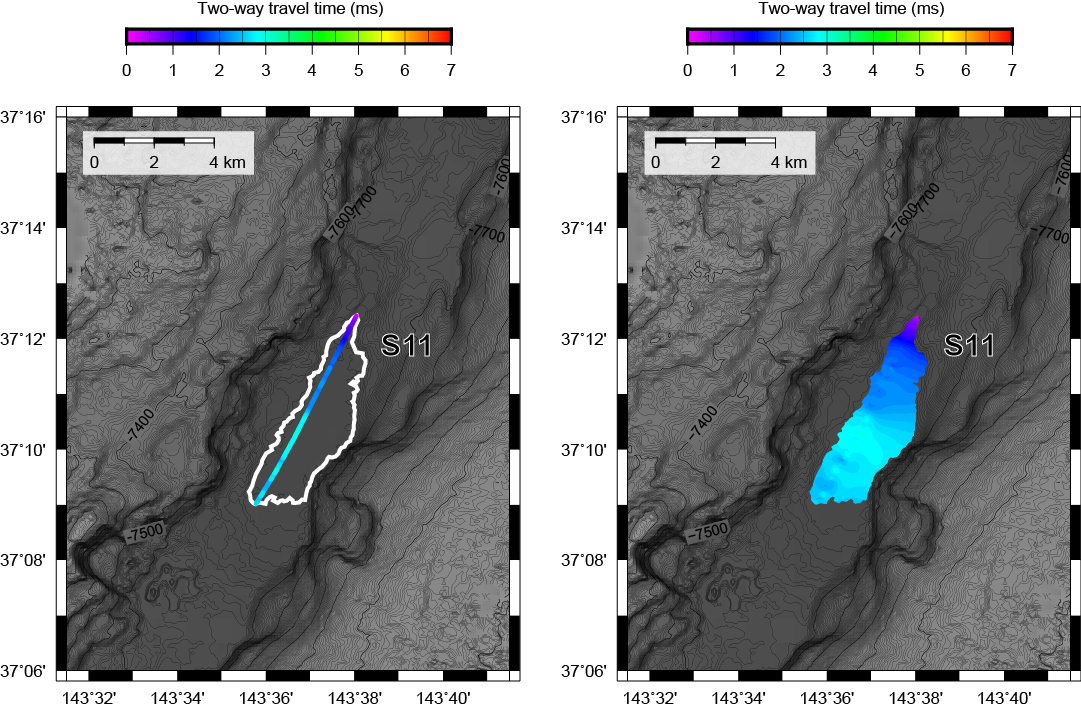


Supplementary Figure 16. (Left) Two-way travel time (TWT) difference between top and base of the 2011 event deposit along the studied SBP lines at the Basin S11 (see Supplementary Figure 4). (Right) TWT-Isopach map of the 2011 event deposit over the entire Basin S11.


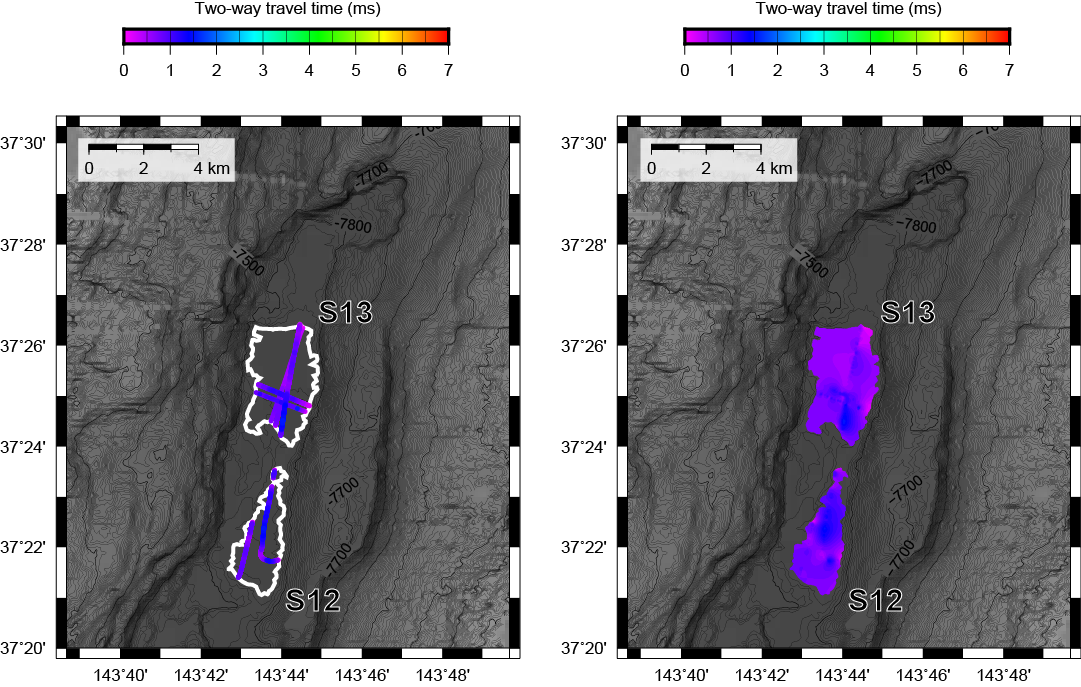


Supplementary Figure 17. (Left) Two-way travel time (TWT) difference between top and base of the 2011 event deposit along the studied SBP lines at the basins S12 (see Supplementary Figure 5) and S13 (Supplementary Figure 6). (Right) TWT-Isopach map of the 2011 event deposit over the entire basins S12 and S13.


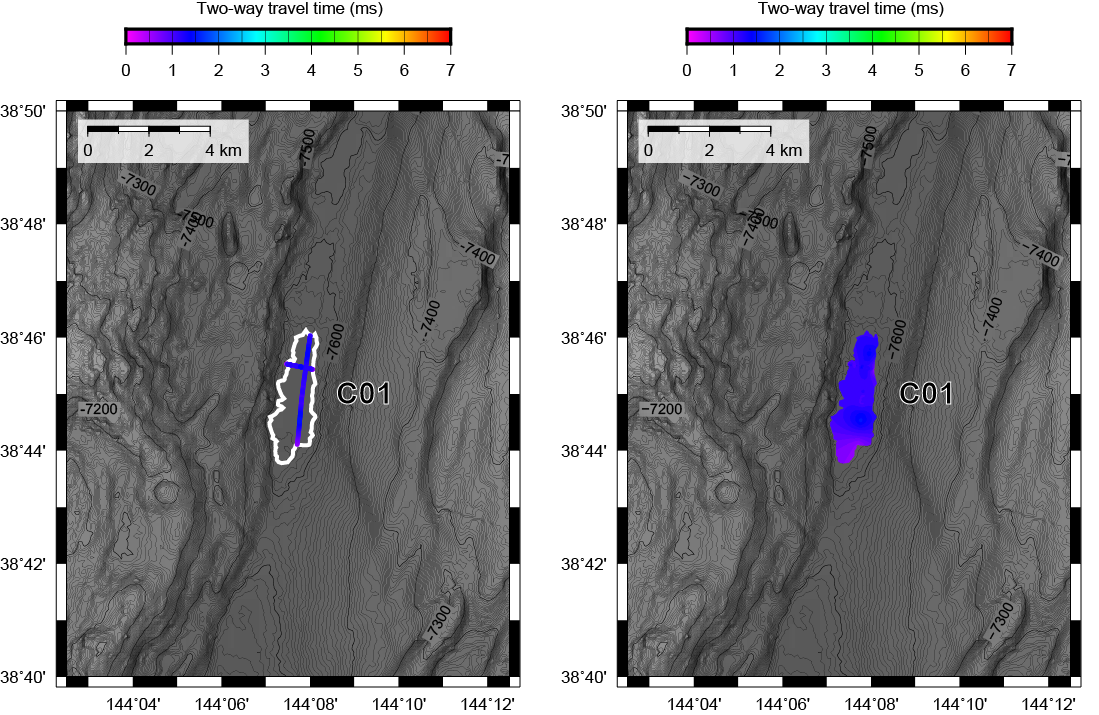


Supplementary Figure 18. (Left) Two-way travel time (TWT) difference between top and base of the 2011 event deposit along the studied SBP lines at the Basin C01 (see Supplementary Figure 7). (Right) TWT-Isopach map of the 2011 event deposit over the entire Basin C01.


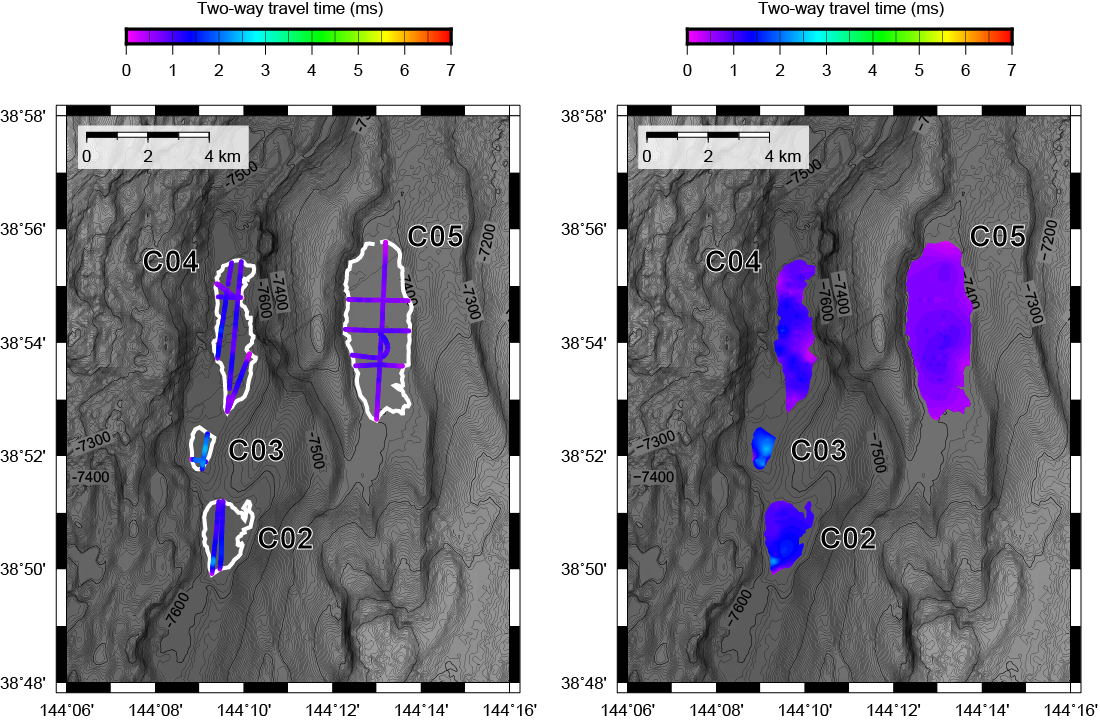


Supplementary Figure 19. (Left) Two-way travel time (TWT) difference between top and base of the 2011 event deposit along the studied SBP lines at the basins C02 (see Supplementary Figure 8), C03 (Supplementary Figure 9), C04 (Supplementary Figure 10), and C05 (Supplementary Figure 11). (Right) TWT-Isopach map of the 2011 event deposit over the entire basins C02, C03, C04, and C05.


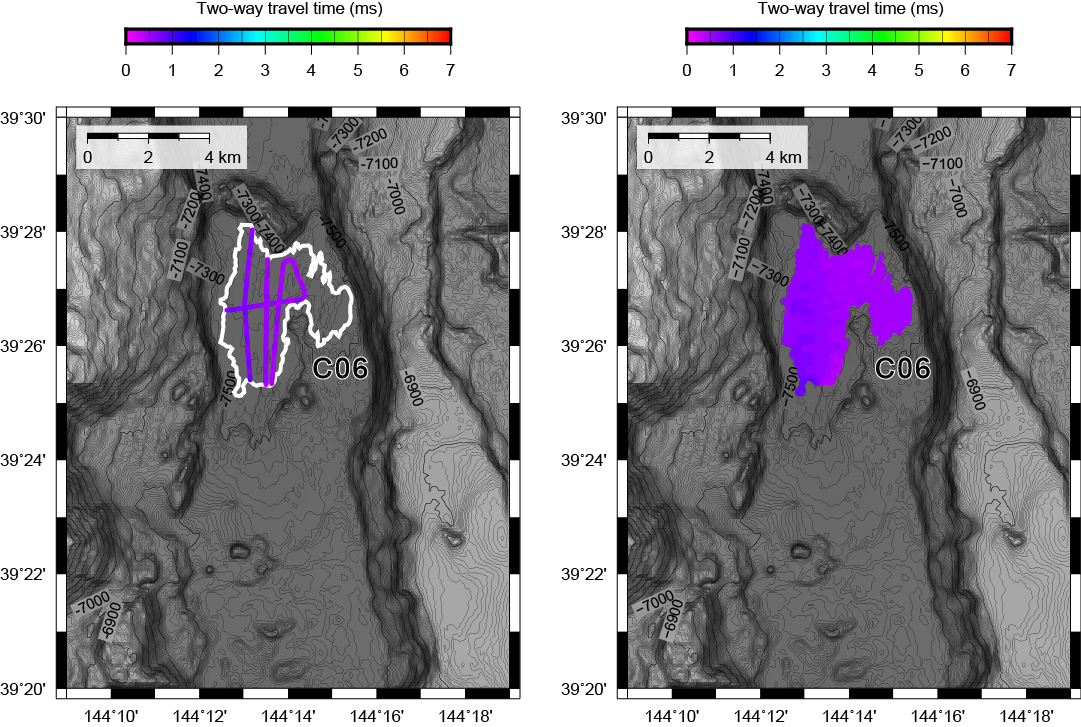


Supplementary Figure 20. (Left) Two-way travel time (TWT) difference between top and base of the 2011 event deposit along the studied SBP lines at the Basin C06 (see Supplementary Figure 12). (Right) TWT-Isopach map of the 2011 event deposit over the entire Basin C06.

Supplementary Table 1. Thickness, areal extent, and volume of event deposit at a given fill-basin in the Japan Trench.

| Basin ID | Lon (°E) | Lat (°N) | Maximum thickness (m) | Area (km^2^) | Volume  (x 10^6^ m^3^) | Reliability |
| --- | --- | --- | --- | --- | --- | --- |
| Southern Japan Trench | | |  |  |  |  |
| S01 | 142.727 | 36.076 | 3.7 (+0.6/-0.1) | 29.4 (+/-0.5) | 54 (+11/-3) | A |
| S02 | 142.731 | 36.100 | 0.8 (+0.2/-0.1) | 0.2 (+/-0.05) | 0.13 (+0.08/-0.04) | C |
| S03 | 142.760 | 36.103 | 1.0 (+0.2/-0.1) | 0.1 (+/-0.04) | 0.07 (+0.05/-0.03) | C |
| S04 | 142.836 | 36.166 | 3.2 (+0.6/-0.1) | 4.1 (+/-0.2) | 9.5 (+2.2/-0.9) | C |
| S05 | 142.861 | 36.194 | 1.3 (+0.3/-0.1) | 0.2 (+/-0.04) | 0.15 (+0.08/-0.05) | C |
| S06 | 142.938 | 36.328 | 4.9 (+0.7/-0.1) | 18.3 (+/-0.4) | 43 (+9/-3) | C |
| S07 | 142.999 | 36.403 | 2.9 (+0.5/-0.1) | 7.2 (+/-0.3) | 12 (+3/-1) | C |
| S08 | 143.103 | 36.535 | 2.7 (+0.5/-0.1) | 1.0 (+/-0.1) | 2.1 (+0.6/-0.3) | C |
| S09 | 143.315 | 36.766 | 2.3 (+0.4/-0.1) | 6.1 (+/-0.2) | 9.3 (+2.3/-1.0) | C |
| S10 | 143.396 | 36.856 | 4.6 (+0.7/-0.1) | 3.2 (+/-0.2) | 12 (+3/-1) | C |
| S11 | 143.614 | 37.171 | 2.2 (+0.4/-0.1) | 10.2 (+/-0.3) | 18 (+4/-2) | C |
| S12 | 143.726 | 37.373 | 1.1 (+0.3/-0.1) | 4.7 (+/-0.2) | 2.9 (+1.0/-0.6) | B |
| S13 | 143.740 | 37.413 | 1.2 (+0.3/-0.1) | 8.2 (+/-0.3) | 4.1 (+1.6/-0.9) | A |
| Central Japan Trench | | |  |  |  |  |
| C01 | 144.130 | 38.743 | 1.1 (+0.3/-0.1) | 3.9 (+/-0.2) | 3.1 (+1.0/-0.5) | B |
| C02 | 144.155 | 38.836 | 1.7 (+0.3/-0.1) | 2.5 (+/-0.2) | 2.2 (+0.7/-0.4) | C |
| C03 | 144.152 | 38.869 | 1.8 (+0.3/-0.1) | 0.7 (+/-0.08) | 0.87 (+0.32/-0.16) | B |
| C04 | 144.158 | 38.904 | 1.3 (+0.3/-0.1) | 4.3 (+/-0.2) | 3.1 (+1.0/-0.6) | A |
| C05 | 144.219 | 38.902 | 0.9 (+0.2/-0.1) | 9.4 (+/-0.3) | 4.3 (+1.7/-1.0) | A |
| C06 | 144.219 | 39.423 | 0.7 (+0.2/-0.1) | 12.0 (+/-0.4) | 5.0 (+2.0/-1.3) | A |
| CU01 | 143.989 | 37.953 | N/A | 2.0 (+/-0.1) | 0.41 (+/-0.03) | N/A |
| CU02 | 144.006 | 38.009 | N/A | 3.9 (+/-0.2) | 0.79 (+/-0.04) | N/A |
| CU03 | 143.990 | 38.022 | N/A | 0.7 (+/-0.08) | 0.13 (+/-0.02) | N/A |
| CU04 | 143.995 | 38.098 | N/A | 2.1 (+/-0.1) | 0.42 (+/-0.03) | N/A |
| CU05 | 144.059 | 38.298 | N/A | 1.8 (+/-0.1) | 0.36 (+/-0.03) | N/A |
| CU06 | 144.125 | 38.576 | N/A | 0.1 (+/-0.04) | 0.03 (+/-0.01) | N/A |
| CU07 | 144.204 | 39.248 | N/A | 23.8 (+/-0.5) | 4.8 (+/-0.1) | N/A |

Reliability: Reliability of data at a given basin. See Section 3 for definition of classes A to C.

Basins CU01 – CU07 are suggested as depositional basin by bathymetry, and have documented 10–20 cm thick 2011-event deposit in sediment cores (e.g., CU02: MR12-E01-PC04 (Ikehara et al., 2016), GeoB16431 (Ikehara et al., 2016; Bao et al., 2018), KS-14-16 PC02 (Ikehara et al., 2018; CU04: MR12-E01 PC03, PC01 (Ikehara et al., 2016) and DC01 (Oguri et al., 2013); CU05: KS-15-3 PC10 (Ikehara et al., 2018); CU06: KS-14-16 PC10 (Ikehara et al., 2018)), although their event deposit thicknesses are not detected through SBP data, either because of small-scale seafloor roughness beyond horizontal resolution of SPB data not to reveal lateral continuous subsurface reflection pattern (e.g., deformation of trench-fill sedimentation by slip to the trench and slumping near the location of CU02 (Kodaira et al., 2012; Strasser et al., 2013)), or the event-deposit thickness is below the vertical resolution of SBP (e.g., for CU07 see also Fig. 1 and 2 in the main text). The volume is thus calculated as a product of the estimated area and thin thickness (20 cm).

Supplementary Table 2. Total organic carbon (TOC) content per area in the surface sediment layer of the hadal trench.

| Name of trench | Water depth (m) | Distance to land (km) | Dry density (kg/m^3^) | TOC at the seafloor (wt%) | TOC per area (kg/m^2^) |
| --- | --- | --- | --- | --- | --- |
| Japan ^a^ | 7410 – 8030 | 170 – 215 | 380+/-50 – 870+/-80 | 1.57 – 2.16 | 4.1 (+3.1/-2.2) –  31.7 (+17.5/-13.8) |
| Mussau ^b^ | 7010 | 255 | 1450 | 0.83 | 1.2 |
| Atacama ^c^ | 7800 | 80 | n/a | 0.64+/-0.25 | 0.6+/-0.2 |
| New Britain ^b^ | 8230 | 55 | 1450 | 0.97 | 1.8 |
| Izu-Bonin (Ogasawara) ^d^ | 9150 – 9260 | 215 | 1760+/-230 | 1.11 | 6.0+/-0.3 |
| Tonga ^d^ | 10800 | 200 | 1320+/-40 | 2.19 | 6.1+/-0.9 |
| Mariana ^e^ | 10900 | 355 | 1260+/-70 | 0.40 | 0.7 – 1.1 |

a: From this study. TOC per area is an integrated value of single event deposit within a given basin by the AD 2011 Tohoku-oki earthquake.

b: From Luo et al. (2018). TOC per area is a depth-integrated value of upper 13 cm.

c: From Danovaro et al. (2003). TOC per area is a depth-integrated value of upper 10 cm.

d: From Wenzhöfer et al. (2016). TOC per area is a depth-integrated value of upper 15 cm.

e: From Glud et al. (2013) and Wenzhöfer et al. (2016). TOC per area is a depth-integrated value of upper 25 cm.

**Supplementary Table 3.** Data of xs^210^Pb from the core GeoB21804.

| Upper (cm) | Lower (cm) | xs^210^Pb (Bq/kg) | 1$\sigma$ (Bq/kg) |
| --- | --- | --- | --- |
| 4.0 | 5.0 | 900.5 | 53.2 |
| 9.0 | 10.0 | 1007.2 | 57.8 |
| 14.0 | 15.0 | 1294.4 | 69.6 |
| 19.0 | 20.0 | 1123.2 | 63.0 |
| 24.0 | 25.0 | 1289.4 | 70.9 |
| 29.0 | 30.0 | 1163.5 | 65.9 |
| 34.0 | 35.0 | 1153.1 | 64.3 |
| 39.0 | 40.0 | 1140.2 | 64.2 |
| 44.0 | 45.0 | 1056.3 | 59.8 |
| 49.0 | 50.0 | 1063.2 | 59.7 |
| 54.0 | 55.0 | 1154.2 | 65.8 |
| 59.0 | 60.0 | 1115.9 | 63.6 |
| 64.0 | 65.0 | 1041.7 | 59.6 |
| 69.0 | 70.0 | 1077.6 | 61.8 |
| 74.0 | 75.0 | 1097.5 | 63.2 |
| 79.0 | 80.0 | 1102.8 | 62.1 |
| 84.0 | 85.0 | 1068.6 | 61.3 |
| 89.0 | 90.0 | 1134.9 | 64.4 |
| 104.0 | 105.0 | 951.7 | 55.0 |
| 119.0 | 120.0 | 905.4 | 49.4 |
| 134.0 | 135.0 | 762.7 | 44.3 |
| 150.0 | 151.5 | 409.8 | 34.3 |
